# Evolution by innovation as a driving force to improve TCR-T therapies

**DOI:** 10.3389/fonc.2023.1216829

**Published:** 2023-09-21

**Authors:** Dolores J. Schendel

**Affiliations:** ^1^ Medigene Immunotherapies GmbH, Planegg, Germany; ^2^ Medigene AG, Planegg, Germany

**Keywords:** adoptive cell therapy, CD40/CD40L interactions, PD-1/PD-L1 inhibition, TCR-T therapy, switch receptors

## Abstract

Adoptive cell therapies continually evolve through science-based innovation. Specialized innovations for TCR-T therapies are described here that are embedded in an End-to-End Platform for TCR-T Therapy Development which aims to provide solutions for key unmet patient needs by addressing challenges of TCR-T therapy, including selection of target antigens and suitable T cell receptors, generation of TCR-T therapies that provide long term, durable efficacy and safety and development of efficient and scalable production of patient-specific (personalized) TCR-T therapy for solid tumors. Multiple, combinable, innovative technologies are used in a systematic and sequential manner in the development of TCR-T therapies. One group of technologies encompasses *product enhancements* that enable TCR-T therapies to be safer, more specific and more effective. The second group of technologies addresses *development optimization* that supports discovery and development processes for TCR-T therapies to be performed more quickly, with higher quality and greater efficiency. Each module incorporates innovations layered onto basic technologies common to the field of immunology. An active approach of “evolution by innovation” supports the overall goal to develop best-in-class TCR-T therapies for treatment of patients with solid cancer.

## Introduction

The starting point for effective immune defense against cancer is the availability of abundant and potent T cells in patients that recognize target antigens on cancer cells and mediate their destruction. From here, additional mechanisms of immune response can be orchestrated by T cells to mobilize other players of the immune system in the fight against cancer. The principle of T cell control of cancer is well established by the success of allogeneic hematopoietic stem cell transplantation (HSCT) in which vigorous T cell responses can cure both lymphoid and myeloid blood cancers (1, 2). Likewise, patients responding to autologous tumor-infiltrating lymphocyte (TIL) therapies demonstrate the capacity of T cells to control solid tumor growth, provide long-term clinical benefit, and even cure some cases of solid cancer (3, 4). These adoptive cell therapies (ACT) rely on naturally occurring T cells in patients that are minimally manipulated to enable their functional activities to unfold against cancer cells. Individual patients can experience great benefit but clinical efficacy of TIL is unpredictable, due to variations in natural immune responses among patients and tumor heterogeneity. Nevertheless, these therapies provide inspiration to develop further ACT that are more widely applicable and deliver more consistent clinical benefit.

TCR-T therapies have the potential to fill the significant unmet medical need for new treatment options for patients with diverse types of solid cancer. Decades of research paved the way to engineer T cells to express a T cell receptor (TCR) that binds to specific antigen expressed on tumor cells and subsequently activates T cells to destroy aberrant cells. In the case of TCR-T cells, the TCR is a recombinant protein (rTCR) engineered for surface expression, but activation occurs through natural intracellular signaling pathways in the recipient T cells that mobilize diverse functions (5, 6). When rTCR are introduced into autologous patient T cells, billions of engineered T cells can be expanded and given back to a patient to deal with large tumor burden.

Pioneering work in use of TCR-engineered T cells for therapy of cancer patients with both blood and solid cancers has demonstrated the power of this approach (7–9). In early studies of blood cancers, ACT using TCR-T cells specific for the *Wilms’ tumor 1* (WT1) antigen were applied post-HSCT in patients with acute myeloid leukemia (AML) and myelodysplastic syndrome (MDS) who were at high risk for relapse using TCR recognizing WT1-derived peptides presented by either HLA-A2 or HLA-A24 allotypes (10–12). In the larger cohort of HLA-A2 patients, Epstein-Barr-Virus (EBV)-specific, patient-derived T cells were used as recipient T cells in order to foster long-term T cell persistence and guard against endogenous TCR causing graft-versus-host-disease (GvHD). Safety and persistence of T cells, without detrimental GvHD mediated by the TCR-T cells, were demonstrated and improved progression free survival and overall survival was projected by comparison with a contemporary matched AML patient control group (11). It should be noted however that GvHD was not seen in the smaller group of HLA-A24 patients who received TCR-T cells derived using CD3-positive peripheral blood T cells (12). The *minor histocompatibility antigen-1* (mHA-1) was used as another target for post-HSCT TCR-T therapy and evaluated in patients with AML (13, 14). A post-HSCT trial in multiple myeloma (MM) patients demonstrated safety and clinical benefit in some patients who received TCR-T cells specific for the cancer-germline line antigen (CGA) *New York esophageal 1* (NY-ESO-1) (15). The CGA *preferentially expressed in melanoma antigen* (PRAME) was identified as a valid target for AML and other blood cancers (16, 17) and was recently evaluated in a phase 1 TCR-T trial in patients with relapsed or refractory AML, MDS and MM^1^ ^,^ ^2^. These studies of first-generation TCR-T therapies demonstrated the important potential of TCR-T therapies to provide clinical benefit to patients with lymphoid and myeloid blood cancers, without major toxicities associated with the TCR-T cells themselves; treatment toxicities were mostly associated with the pre-conditioning regimens of patients or use of interleukin 2 (IL2) to drive TCR-T expansion *in vivo*. Future developments now build upon this foundational work to improve TCR-T therapies for blood cancers, in particular those malignancies that are not adequately addressed with chimeric antigen receptor-T (CAR-T) cell therapies that are remarkably effective in the treatment of B cell malignancies and MM (18).

The first-in-human TCR-T clinical trials in solid cancer followed the footsteps of TIL therapies and TCR were selected for specificity based on clinically relevant responses seen with TIL therapy of melanoma patients, initially including the *melanoma antigen recognized by T cells 1* (MART-1) and melanoma *glycoprotein 100* (gp100); both are differentiation antigens present in pigmented cells, like melanocytes, but are highly over-expressed in melanomas (19). While clinical benefit was seen using TCR-T cells, toxicities directed against healthy tissues also expressing these antigens were seen (20–22). Similar toxicities directed against healthy tissues were found applying TCR-T therapy specific for the *carcinoembryonic antigen* (CEA) (23). These signs of toxicity moved the field to consider other antigens with better safety profiles, such the CGA NY-ESO-1 and *melanoma-associated antigen* (MAGE) gene family members (24). Patients with melanoma and synovial sarcoma receiving TCR-T therapy specific for NY-ESO-1 did not show toxicity for healthy tissues and clinical benefit was seen in some patients, establishing this as a valid antigen for these indications (25, 26). In contrast, MAGE-A3-specific TCR-T therapies caused lethal toxicity related to TCR recognition of identical or similar target epitopes (27–29), demonstrating the critical relevance of target antigen selection, alongside careful vetting of TCR safety profiles. Exciting developments are now underway evaluating TCR-T therapies specific for tumor mutations, like KRAS or TP53 proteins, which are highly prevalent in different solid cancers with high unmet medical need (8, 9, 30).

Armed with preliminary results regarding safety and dangers, benefits and limitations, there has been dramatic growth in clinical trials using TCR-T therapies in the past several years. A recent review covers ongoing trials and results reported for TCR-T trials now in progress or completed (7). Most trials today are still in early stages, with a preponderance of phase 1 studies ongoing at this time, whilst two TCR-T therapy trials are considered pivotal studies. The SPEARHEAD-1 trial investigates afamitresgene autoleucel, which is specific for the MAGE-A4 target antigen^3^. At the time of writing, this TCR-T therapy is currently being evaluated by the FDA for the treatment of synovial sarcoma and, if approved, will mark a major step in providing an additional treatment modality for patients. In addition, the IGNYTE-ESO clinical trial of letetresgene autoleucel targets the NY-ESO-1 antigen in synovial sarcoma and myxoid/round cell liposarcoma (31). Importantly, the FDA approved the first TCR-based therapy in early 2022 for treatment of metastatic uveal melanoma (32). KIMMTRAK is not an ACT but rather is a bi-specific T cell engager (TCE) protein that bridges T cells to tumor cells through a specific antibody domain that binds and activates CD3 on normal T cells of patients while it uses a melanoma-specific TCR binder recognizing gp100 that is highly expressed on metastatic uveal melanoma cells. Most TCR-T therapies use autologous patient-derived T cells for generation of TCR-T therapies, but developments using allogeneic T cells and induced pluripotent stem cell (iPSC)-derived T cells are ongoing (8, 33, 34), as well as use of other cell types such as natural killer (NK) cells and gamma-delta T cells that express TCR binders to direct tumor cell recognition, as reviewed extensively elsewhere (30, 35). Important advances in TCR-T therapies can be expected in the future using next generation approaches.

**Figure 1:**
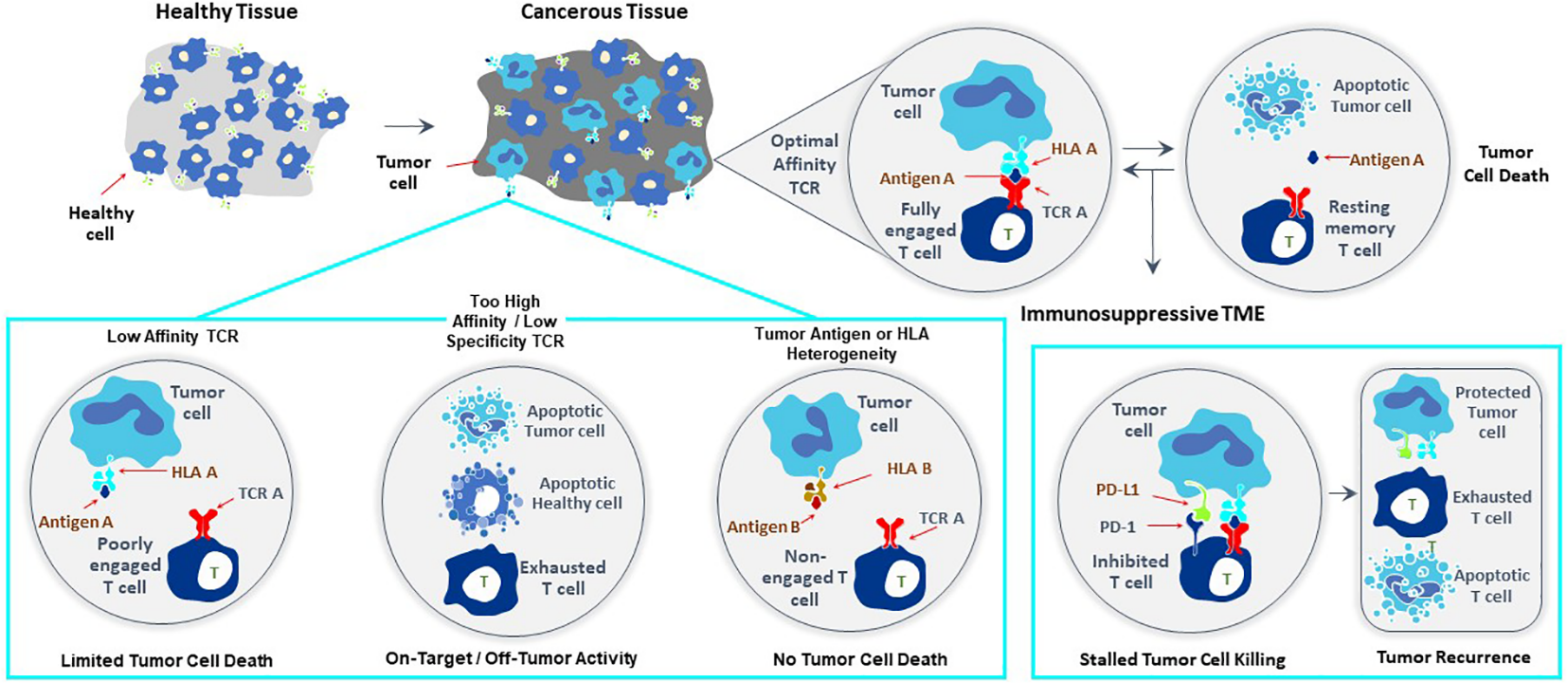
Major challenges to improve clinical efficacy of TCR-T therapies for solid cancer. Three challenges are major impediments to success of TCR-T therapies: affinity of the TCR that recognizes a peptide-HLA complex on tumor cells, heterogeneity in peptide or HLA expression on tumor cells that can diminish TCR recognition of tumor cells allowing their escape from TCR-T cell attack and mechanisms mobilized by tumor cells, including expression of PD-L1 that interacts with PD-1 on TCR-T cells and inhibits their functions. Singly and in combination these three parameters strongly impact the efficacy of TCR-T therapy and represent critical challenges that need to be met in next generation TCR-T therapies to improve treatment outcomes for patients with solid cancer.

T cells recognize targets originating from both surface and intracellular proteins, opening the door for treatment of diverse forms of cancer with TCR-T therapies, making this form of ACT especially attractive for therapy of solid cancer. The frequent and dramatic responses achieved with CAR-T therapies for blood cancers have not been seen to date with TCR-T therapies of solid cancer (5, 6). If one closely examines the current state of TCR-T clinical development and challenges for success, several points are readily apparent as touchstones where relevant steps can be taken to develop next generation TCR-T therapies with better attributes for use against solid cancer (**Figure 1**). CAR-T cells use antibody binding domains to interact specifically with surface antigens on tumor cells and affinities of antibody-based binders are far higher than those of natural TCR present in T cells of most patients or healthy donors (5, 6). Thus, first and foremost comes the need to use TCR that are of higher affinity to improve efficacy, but with the understanding that affinity dictates not only sensitivity of target detection but also specificity, which are interdependent parameters that are of paramount importance for safety (7–9). A TCR needs to recognize tumor cells with great discrimination, being highly specific for the target on tumor cells, while not recognizing healthy tissues. This is particularly critical since TCR-T cells remain active and present for long time periods, even years, after infusion into patients. At the same time, the TCR needs to be sensitive enough to recognize tumor cells that express only low levels of target antigen. This is best achieved if the TCR is of high affinity. However, a high affinity TCR may be less specific, losing the ability to fully ignore healthy tissues, either through “on target”/”off tumor” recognition or through “off target”/”off tumor” cross-recognition of healthy cells, dependent on the nature of the target antigen and the TCR. Thus, the task is arduous to find TCR with “optimal” affinity for any selected target antigen that fulfill the necessary requirements for specificity, sensitivity and safety, and such TCR are rare. If an optimal TCR is found for TCR-T therapy development, the next impediment for clinical success lies in tumor cell heterogeneity for TCR target antigen expression, which leads to failure of the TCR-T cells to eliminate all tumor cells. TCR-T cells themselves can even select for tumor cell variants that cannot be recognized by their specific TCR. This problem can only be overcome if TCR-T therapies display a plethora of functional capacities that allow them to infiltrate tumors, recruit other cells and orchestrate fulminant responses needed for tumor control. Combinations of TCR-T cells recognizing different target antigens and different human leukocyte antigens (HLA) can be deployed to deal with tumor heterogeneity and wider population diversity, as discussed below. Thereafter, the tumor microenvironment (TME) of many solid cancers creates special challenges for TCR-T cells to orchestrate complex antitumor responses to achieve better clinical results (8, 9). In particular, tumor cells themselves and other cells they recruit to the TME can negatively regulate the function of TCR-T cells and other cells of the immune system, protecting tumors from direct T cell attack but also rapidly halting the ability of TCR-T cells to mobilize the fulminant immune responses involving additional cell types needed to improve clinical efficacy.

To advance TCR-T therapies with better efficacy, durability of response and safety, an end-to-end platform was established that addresses these diverse technological challenges at each step of development, from target antigen identification, to isolation of TCR, through to delivery of therapies to patients. Primary emphasis is placed on generating TCR with optimal affinities and expressing them in recipient T cells that are further engineered to mobilize diverse antitumor immune responses, as needed to fight solid cancers in the challenging setting of an immunosuppressive TME. A secondary focus to address challenges of tumor heterogeneity and population diversity is the creation of a “Library” of multiple drug products that have the ability to target different antigens, different HLA and combined with different technologies to mitigate the wide ranging immunosuppressive effects of the TME.

## End-to-end platform for TCR-T therapy development

**Figure 2:**
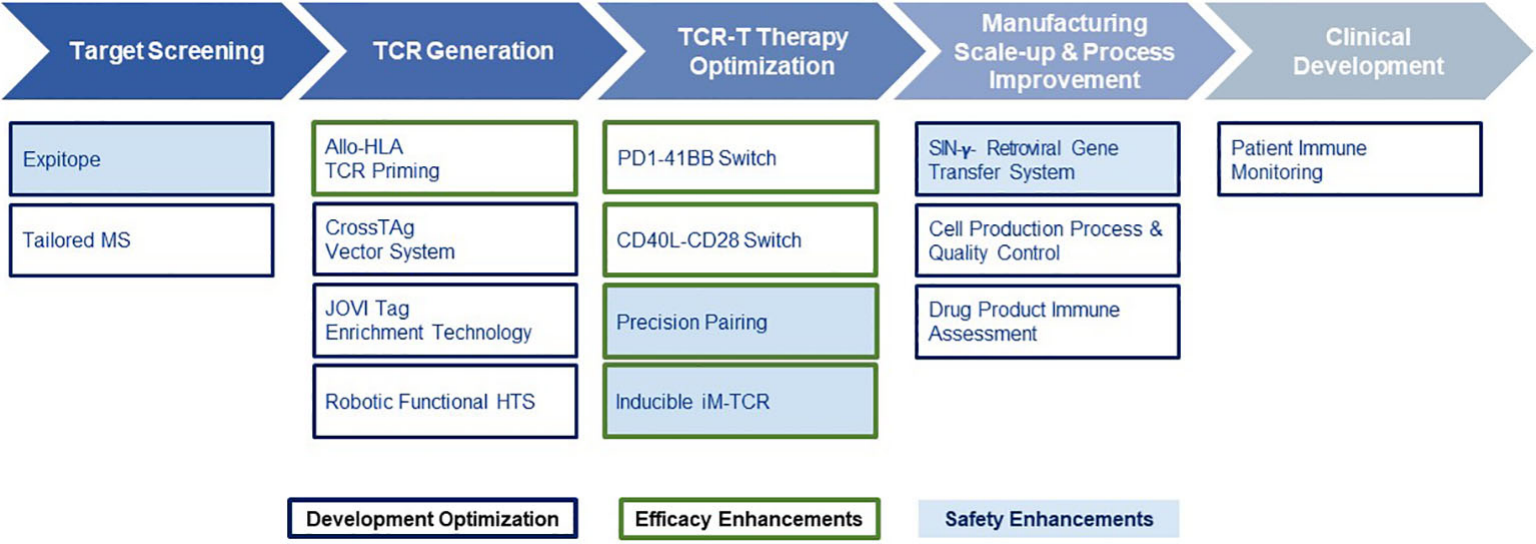
End-to-End Platform for TCR-T Therapy Development. The E2E Platform consists of five modules that cover important steps to generate TCR-T therapies, including Target Screening, TCR Generation, TCR-T therapy Optimization, Manufacturing Scale-up & Process Improvement and Clinical. Specialized innovations are embedded in each module that provide tools and technologies as unique solutions for challenges in TCR-T therapy development. The associated innovations discussed sequentially in the text for the first three modules are listed below each module. Technologies that contribute to *Product Enhancement* are framed in green. These are designed to enable TCR-T therapies to be more specific, safer, and more efficacious. Innovative technologies that serve for *Development Optimization* are framed in dark blue. These enable discovery and development processes for TCR-T therapies to be performed more quickly, with higher quality and greater efficiency. Innovations that contribute to the safety profile of TCR-T therapies are colored in blue.

Given the multiple technological challenges associated with developing specific, sensitive and safe TCR-T therapies that can best address the unmet efficacy and safety needs for patients with solid tumors, we have assembled five discrete R&D modules consisting of individual technologies that form a continuous “End-to-End Platform” for the production of best-in-class TCR-T therapies. The technologies in these modules have been built on knowledge and basic techniques common to the field of immunology, which have been reviewed separately in depth and to which frequent reference will be made in this overview (7–9). Importantly, unique innovations have subsequently been added to either deeply enrich and improve an individual module, or new technologies have been developed *de novo* as depicted in **Figure 2**. The five modules of the E2E Platform span preclinical and clinical TCR-T therapy development: *Target Screening, TCR Discovery, TCR-T Therapy Optimization, Manufacturing Scale-up & Process Improvement and Clinical Development.* This overview describes several innovations that are embedded in the first three preclinical modules. The two clinical modules that cover drug product manufacture and clinical development are not considered here, as these are highly specialized and change continually for different clinical studies. However, first generation methods used to manufacture and characterize TCR-T drug products, as well as tools designed for immune monitoring of treated patients, were applied in a multi-center Phase I TCR-T therapy trial of patients with relapsed/refractory AML, MDS and MM (NCT03503968). A success rate of 92% was achieved in manufacture of TCR-T cells using starting cells of heavily pre-treated patients. Fit-for-purpose immune monitoring tools enabled TCR-T cells to be specifically tracked and allowed detection of transferred T cells early after start of treatment and also at one year in a patient showing long-term clinical benefit. Specific information on these results is available online^1^ ^,^ ^2^. E2E Platform technologies to date are centered on classical CD8 T cells for development of TCR-T therapies for solid cancers but they can also be used for blood cancers and applied to CD4 T cells, with some modifications. Other types of T cells, including gamma-delta T cells, express unique types of antigen receptor used for treatment of cancer. They are not integrated in the E2E Platform but their use in ACT has been recently reviewed (35).

The founding premise underpinning our approach of “evolution by innovation” for the E2E Platform is to hone closely to the natural structures, natural signaling pathways, and natural cell functions that have evolved over millions of years for T cells. As TCR-T cells are engineered to develop best-in-class therapies for patients, an attempt is always made to stay within the individual evolutionary constraints that nature has placed on T cells, such as levels of TCR affinity and T cell avidity, spatial orientation of receptors with respect to how they interact with each other on the surface of T cells and, equally important, how they interact with their corresponding ligands on other cells in the complex microenvironment encountered by TCR-T cells *in vivo*. Although cutting-edge synthetic biology is employed to refine functions in T cells, the modifications follow very well-known signaling pathways and functions that are well characterized and naturally utilized in T cells. As far as possible, the changes made retain the intracellular regulatory pathways that naturally restrain over-reactions of T cells. In this way, the final aim to achieve improved clinical efficacy is combined with maintenance of safety for TCR-T therapies developed through the E2E Platform.

## Module 1: Target antigen selection

**Figure 3:**
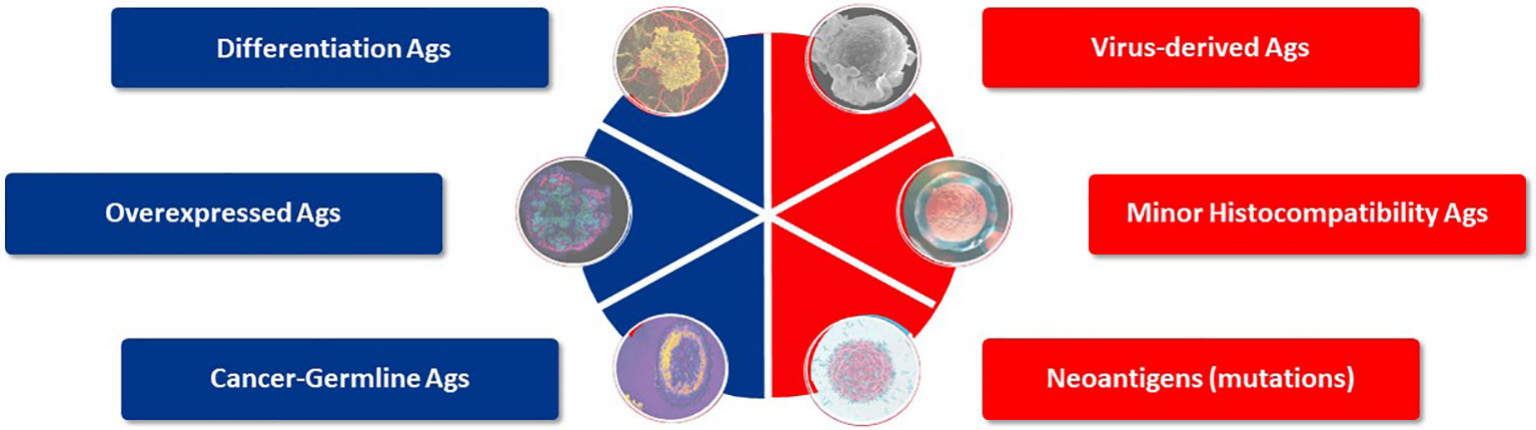
Target antigens suited for TCR-T therapy. Six categories of potential target antigens for T cell based immunotherapies have been identified. Three groups are derived from self-proteins (marked in blue) and three groups derive from foreign proteins (marked in red). Multiple antigens are clustered in each group and examples of validated targets for ACT are described in the text. Target antigens for cancer can also be characterized as tumor-associated antigens (TAA), which show prominent expression in tumors but do have counterparts in healthy tissues, whereas tumor-specific (TSA) antigens are limited to exclusive expression tumor cells. Cancer-germline antigens are examples of TAA and mutated proteins found in cancer cells represent TSA.

Target antigen selection is the starting cornerstone for TCR-T therapy to treat any single indication or group of cancers. Historically, six general categories of antigen have been described that may provide suitable targets for TCR-T therapies of cancer (7–9) (**Figure 3**). These categories of tumor antigen can be viewed in two ways: they can be classified as *self-versus-foreign* antigens or as *tumor-associated-versus-tumor-specific* antigens. Many individual protein target molecules are encompassed in each category and each candidate antigen requires deep assessment to establish its suitability for use in clinical development and inclusion in clinical trials to establish validity as a good target for TCR-T therapy, as reflected in the examples of TCR-T trials described above. The *self-versus-foreign* dichotomy strongly impacts the affinities of TCR that can be isolated for a given antigen, as central tolerance to self-proteins limits TCR affinity but does not impact sensitivity for foreign antigens, as discussed in detail in Module 2. Differentiation antigens, as self-proteins, can provide specificity for tumors of particular tissue origin but safety can be impacted by attack of healthy tissues that also express the target, as seen with MART-1 as the target for TCR-T therapy of melanoma (21, 22, 36). Universal antigens, like telomerase and survivin, are self-proteins often over-expressed in tumor cells and judged to have wide application because high proliferation requiring telomerase and avoidance of apoptosis through expression of survivin are hallmarks of cancer (37). On the other hand, these characteristics are also hallmarks of activated T cells and we have shown that TCR-T cells specific for survivin can cause T cell fratricide (38), raising critical issues for use as a target antigen. MHA are genetic polymorphisms that differ between two allogeneic individuals and thereby belong in the category of foreign antigens. They can be safely used as targets if their expression is restricted to cells of the hematopoietic lineage, limiting use to patients who undergo allogeneic HSCT that provides resistant stem cells to the recipient, which cannot be recognized by mHA-specific TCR-T cells (13, 14). Viral antigens, as foreign proteins, can be well suited targets for those cancers arising as a result of viral persistence and if the viral target proteins are retained and well expressed in the relevant cancer indications. Residual expression of viral proteins in some healthy tissues carries risk of “on target”/”off tumor” attack, as could be the case in virus-driven hepatocellular carcinoma (39).

Among these antigen categories, the group of CGA, as self-proteins over-expressed in tumors but with highly restricted expression in healthy tissues, and neoantigens, as foreign antigens arising as mutations restricted to tumor tissues, form the basis of most TCR-T cell therapies under current clinical development (7). CGA represent tumor-associated antigens (TAA) since they have counterparts in healthy tissues, while neoantigens are tumor-specific antigens (TSA) since their expression is limited to tumor cells. Mutations can be specific for tumors of individual patients or commonly shared by tumors of various type and present in different individuals, such as KRAS mutations (36). There are also a few antigens deriving from diverse sources such as endogenous retroviruses or non-coding gene regions, or based on detection of lipids or other non-protein targets that can also serve as targets for quite unique T cells (8, 9); some are even considered to be candidates for universal targets for cancer therapy (33).

Several approaches have been used to validate target antigens relevant for TCR-T therapy. The oldest approach uses TIL themselves to define the TCR target ligands they recognize and has successfully defined several classes of clinically relevant TAA (3, 4, 8, 40). Currently, this approach is strongly supported by next generation sequencing (NGS) of tumor cells to identify TSA that are often the targets of effective TIL responses (8, 41, 42). Large scale mass spectrometry (MS) of cancer specimens is a second approach used to define targets displayed by tumor cells that are not found on healthy tissues (8, 43–45). Because many thousands of targets are found with MS studies, artificial intelligence is now implemented to help select candidates for experimental T cell validation (8, 9, 44–46). A third approach relies on *in silico* and *in vitro* tools to identify target antigens and predict target epitopes for TCR (8). With this approach, functional screens are implemented that evaluate T cell responses to specific target ligands predicted *in silico* rather than relying on extensive searches for antigens and peptides by NGS or MS. T cell responses of healthy donors can be used to confirm the relevance of predicted TCR ligands, bypassing the need for TIL or patient peripheral blood T cells to validate target antigens.

The *Target Screening* module of the E2E Platform follows the third approach for target antigen identification and combines *in silico* and *in vitro* methods to match target antigens to cancer indications, understand how target antigens are expressed in individual cancers and determine the profile of antigen expression in healthy tissues, as a critical safety assessment. RNA and protein expression of candidate antigens are assessed in cancer specimens and healthy tissues, as well as in tumor cell lines needed for later experimental studies. Particular emphasis is placed on evaluation of the actual target ligands of TCR that are recognized on tumor cells and initiate T cell responses.

**Figure 4:**
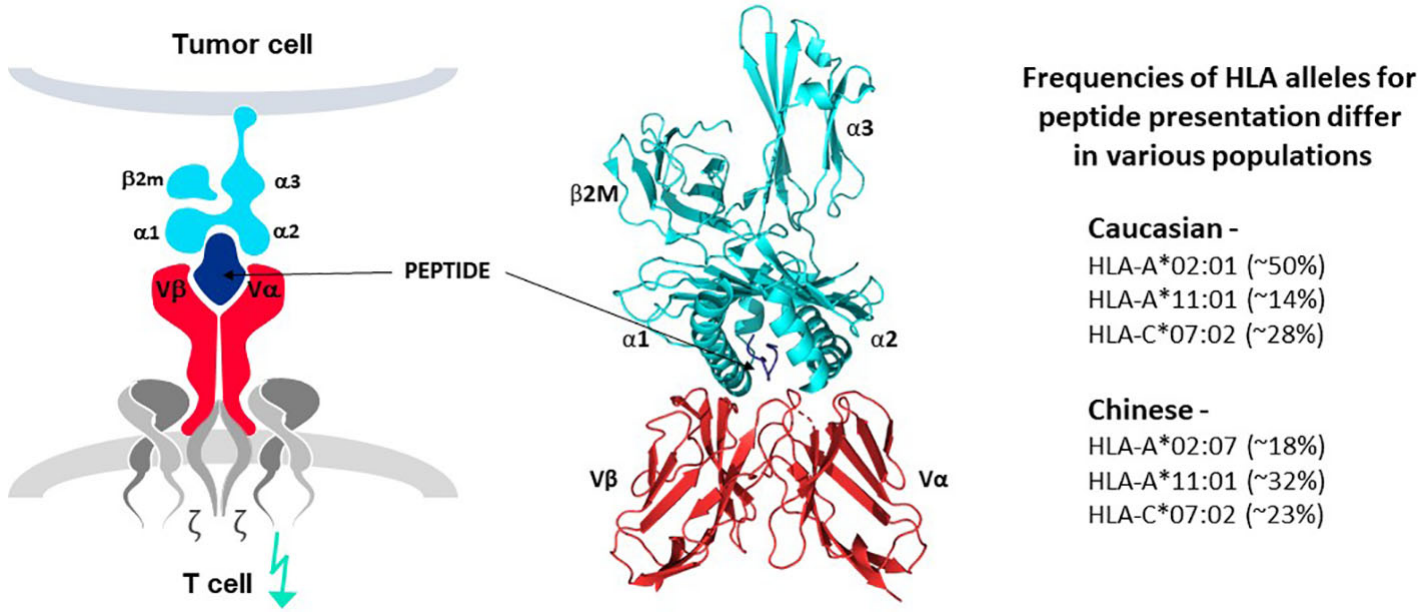
TCR-peptide-HLA interactions. TCR heterodimers (red structures in left and middle panels) recognize antigen in the form of a peptide derived from the target antigen that is presented by one of the HLA allotypes of the target cell (peptide in dark blue and HLA molecule in bright blue, left and middle panels). Each HLA allotype has a unique peptide binding groove that accommodates different peptides that share some complementary features but each single HLA molecule will bind only one peptide, forming the pHLA complex that interacts with a single TCR. TCR-associated CD3 proteins and zeta chains (grey structures, left diagram) transfer signals for T cell activation upon TCR-pHLA binding. Three dimensional models of this interaction reveal that the TCR makes contact with both the peptide and HLA, demonstrating that the ligand for the TCR is comprised of both components of the pHLA complex (ribbon structure in middle panel). HLA allotypes are encoded by diverse HLA alleles that vary in frequency in major world populations (right-hand list). [Software for image preparation: The PyMOL Molecular Graphics System, Version 2.5.4 Schrödinger, LLC].

The principle of human leucocyte antigen (HLA)-restricted peptide presentation governs the ability of TCR to recognize target epitopes derived from intracellular and extracellular proteins and initiate T cell responses. This is alternatively known as Major Histocompatibility Complex (MHC) restriction to cover the general principle of TCR recognition in several species. TCR interact with antigen-derived peptides that are presented on target cells by an HLA protein, in the form of a peptide-HLA (pHLA) complex, as illustrated in **Figure 4** (7–9). HLA proteins encoded by different HLA alleles are designated as HLA allotypes and each individual has multiple MHC class I and class II alleles that encode their different HLA allotypes. Each HLA allotype has a unique peptide binding groove that accommodates different peptides that share some complementary features (46–49), but each single HLA molecule will bind only one peptide, forming the pHLA complex that can interact with a single TCR (50). Many thousands of different pHLA complexes can be expressed on the surface of a cancer cell, based on the entire proteome of the cell as a source of peptides and the different HLA allotypes of the patient, as revealed by MS studies (8, 46–49). Most pHLA complexes will be ignored by T cells due to immune tolerance for peptides originating from normal cellular proteins, so-called self-peptides. Some pHLA complexes, however, can mark cancer cells for T cell recognition, as their peptides are different from those of healthy tissues. For example, peptides originating from mutations or from proteins that are re-expressed or over-expressed in tumor cells can serve as markers for TCR recognition, as discussed above.

For safety reasons, any selected target protein matched to any cancer indication(s) should ideally be well expressed in the cancer (“on tumor”) but missing in healthy tissues (“off tumor”). Exceptions are made (19), as with the gp100 protein of KIMMTRAK targeted in uveal melanoma, which is also present in normal melanocytes. Here the option to treat a deadly cancer (“on target”/”on tumor”) was presumably deemed more beneficial than the risk for attack of healthy tissues (“on target/”off tumor”) and fortunately this was shown to be the case through improved overall survival of patients (32). This is also the consideration for CGA, also designated as cancer-testis antigens (CTA), which are well expressed in various tumors but also found in testes, ovaries and placenta (51–53). Severe “on-target”/”off-tumor” toxicity has not been frequently observed (7–9), which would otherwise strongly limit TCR-T therapy development using CGA to target solid cancers.

Specific pHLA expression by cancer cells is also critical for target antigen selection. The HLA allotypes of a patient dictate which peptides of a target protein will be presented on the cancer cell surface. Class I HLA allotypes, encoded by the HLA-A, -B, and -C alleles, form pHLA complexes that bind to TCR of CD8 T cells. Class II HLA allotypes, encoded by the HLA-DR, -DQ and -DP alleles, form pHLA complexes that bind to TCR of CD4 T cells (8). Currently, TCR-T specificity usually encompasses a pHLA target ligand for CD8 T cells that uses a common class I HLA allotype for two practical reasons: more patients can be treated and these target ligands are expressed constitutively by somatic cells that give rise to most solid cancers. HLA class II allotypes are only expressed constitutively by cells of the hematopoietic lineage and are better suited for peptide presentation for blood cancers. Fortunately, HLA class I molecules are expressed by blood cancers so that class I HLA allotypes can provide peptide presentation for most types of cancer (7, 8).

### Tools for target antigen assessment *in silico* and *in vitro*

Many *in silico* tools for general antigen characterization utilize open access databases as rich sources of information to identify potential target antigens *in silico*, based on extensive data of RNA and protein sequences found in many types of cancer, in cancer cell lines and also in healthy tissues. These include the TANTIGEN, CAD and IEDB data collections as examples (54–56). Critical information regarding potential target expression in healthy tissues also draws on a wealth of open access data, such as ProteomicsDB, ExpressionAtlas, and PAXdb (57–59), which continually expands to include more tissues and samples. RNA and protein expression in healthy tissues can be examined using the GTEx (60) and Human Protein Atlas (61) databases and *in silico* programs are available to analyze this information (62). The CCLE database (63) provides important information on antigen expression in diverse cancer cell lines, with additional cell lines included in the TCLP database (64). This information is particularly useful to select cell panels for *in vitro* studies, as many characterized cell lines are available from cell repositories, like ATCC^4^ and ECACC^5^. MS data of pHLA complexes is continually expanding and immunopeptidome databases catalog actual pHLA complexes that can also be considered in antigen selection (65, 66).

Commonly available *in vitro* methods are used to further validate target antigen expression. The *Target Screening* module of the E2E Platform incorporates NanoString^6^ RNA assessment to compare defined groups of target genes in tumors and healthy tissues, qPCR specific for individual targets, cDNA arrays of cancer samples and healthy tissues, including those specialized for brain tissues, as well as Western blots and immunohistochemistry of specific target proteins in cancer specimens.

### Selection and safety assessment of pHLA complexes *in silico*

*Expitope* is an innovative *in silico* web-server integrated for use in the first two modules of the E2E Platform, which is continually improved to identify pHLA complexes as targets for TCR. This web-tool determines the interactions of peptides derived from a target gene or protein sequence for their potential to bind to a given HLA allotype (67–69). Parameters related to pHLA complex formation can be set with different thresholds, allowing assessments to be stratified for stringency. For example, a threshold for pHLA binding affinity, designated as the MHC Score, can be varied to restrict peptide assessment to those predicted to bind to a selected HLA allotype with high affinity. A Composite Score encompasses parameters of proteosome processing of a peptide from a full length protein (Clevage Score), transport of the peptide into the endoplasmatic reticulum (ER) where it can bind to an HLA class I allotype (TAP Score) and the MHC Score (69–72), adding further information on how well a pHLA complex is predicted to be generated from a target protein and presented at the cell surface. Other *in silico* programs in the field can provide similar information derived from various dispersed sources, but an important advantage of this web-server is that it provides continual updates, giving access to new sequence data as it becomes available online. This dynamic and inclusive property of Expitope allows pHLA complex expression to be assessed in full alignment with the most recent information on genome and proteome sequences which are integrated in one web-tool for simple and efficient use (69).

**Figure 5:**
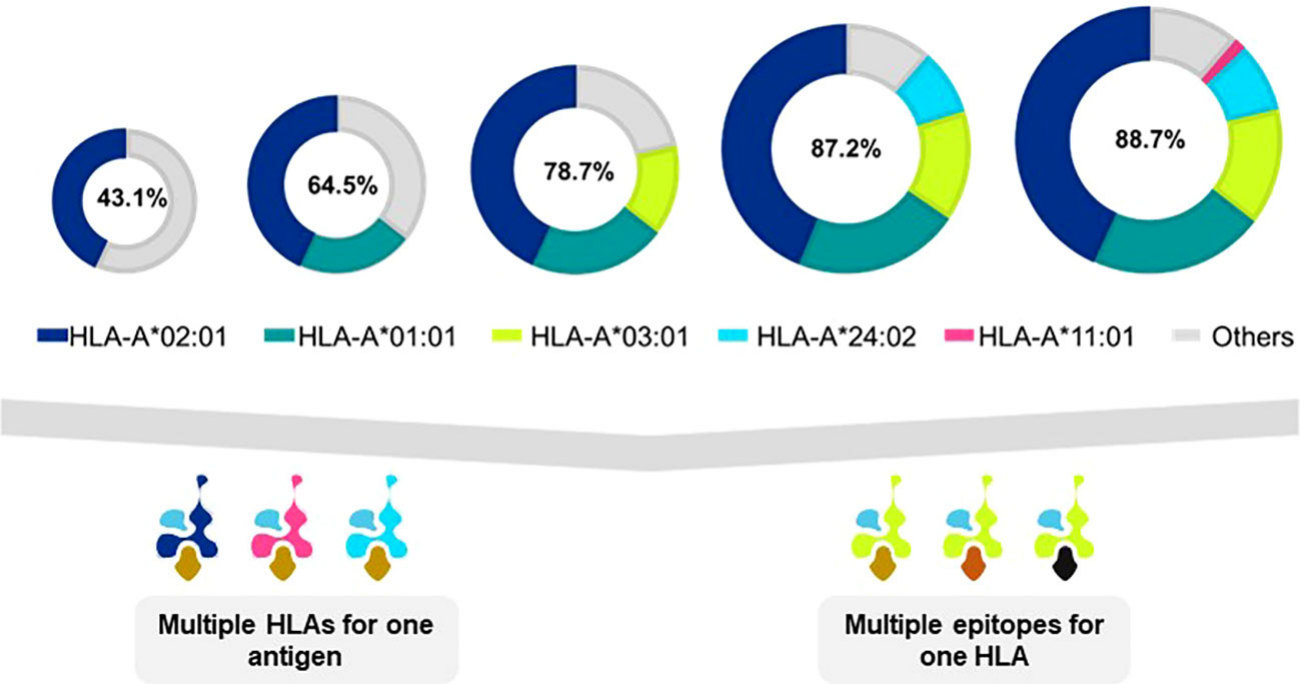
Antigen presentation by five HLA-A alleles expands TCR-T therapy to HLA diverse patient populations. The frequencies of HLA alleles that encode different HLA allotypes vary in different world populations. As individual TCR-T therapies are restricted by different HLA allotypes, the need arises to develop TCR-T therapies to meet the needs of HLA diverse patient populations. One approach to achieve this goal is to develop TCR-T therapies that use five common HLA-A allotypes that cover almost 90% of individuals in Germany^x^. TCR-T therapies based on TCR recognizing pHLA complexes with prevalent HLA allotypes will dramatically expand the scope of treatable patients (upper diagram). A single target antigen may provide peptide sequences that can bind to several HLA allotypes (lower left diagram). Alternatively, several different antigens can provide peptides for a given HLA-A allotype to yield TCR-T therapies for shared indications (lower right diagram). These two approaches may be combined to develop a cluster of TCR-T therapies that can address the broad medical needs of patients worldwide.

Many TCR-T therapies in clinical study currently use TCR that recognize peptides presented by the HLA-A2 allotype, encoded by the HLA-A*02:01 allele, which is the most frequent HLA allele in the US and northern European populations (73). Other trials use TCR restricted by HLA-A*11:01- and HLA-A*24:02-encoded molecules as HLA allotypes frequent in Asian populations (74). As clinical studies progress and validated target antigens are identified for specific cancers, treatments will be needed for additional patient populations. TCR-T therapies based on TCR recognizing pHLA complexes with other prevalent HLA allotypes will dramatically expand the scope of treatable patients, as illustrated for one group of HLA-A alleles in **Figure 5** (upper) that were identified in a specific screening study of patients in Germany^7^. It is unlikely that a single target antigen will carry five peptide sequences that bind sufficiently well to these five prevalent HLA-A allotypes to yield a complete set of pHLA ligands from a single target antigen. However, peptides derived from a few target antigens matched to the same indication(s) could provide pHLA complexes as targets for TCR-T therapies that would cover many more patients with diverse HLA alleles (**Figure 5** lower). To this end, Expitope allows pHLA interactions to be studied for over 100 HLA class I alleles, with the known universe of RNA sequences and proteins, so that TCR-T pipeline strategies can be considered for patient populations worldwide. Expitope is available to the scientific community as an open access web-server^8^.

The Expitope program also embodies safety screens of diverse pHLA complexes. It can search for expression of a specified pHLA complex in more than 20 healthy human tissues, and subtypes thereof as new data becomes available, and quantify levels of expression based on frequency of RNA transcripts found in tissue specimens (67–69). These safety screens can pinpoint healthy tissues that could theoretically be recognized due to “on-target”/”off-tumor” expression and require careful assessment and de-risking to assure safety as a target ligand for TCR-T therapy. If Expitope safety screens are used in early stages of antigen selection, problematic pHLA epitopes can to be avoided, as for example peptide sequences shared by members of homologous gene families, some of which can be expressed in healthy tissues and lead to lethal cross-recognition (28).

Expitope is also used to identify mismatched peptides that differ from the wild-type target peptide by up to 50% replacement with any other amino acid, as well as assess their presence and prevalence in healthy tissues. Cross-recognition of a mismatched peptide led to patient deaths in clinical trials of TCR-T therapy due to expression of a cross-reactive epitope in heart tissue (75); this cross-reactivity between MAGE-A3 and titin could be identified with Expitope. While a mismatched peptide sequence may be predicted *in silico* based on mRNA or protein searches of healthy tissues, this does not mean it is processed or presented as part of a pHLA complex (76) or at a level that can be detected by a TCR-T cell. Thus, functional experiments are critical to establish the safety profile of any pHLA complex and its corresponding TCR, as one of the principal features for any specific target-TCR combination selected for TCR-T therapy.

In the end, the final selection of pHLA complexes that are well suited ligands for TCR-T therapies requires extensive functional evaluation of TCR-T cell recognition of cancer cell lines and patient cancer specimens, as available. Certainly, TCR-T cells should not recognize healthy tissues and this can be tested through direct assessment of recognition of cell lines and fresh healthy tissues, modified to express the relevant HLA allotype. Combined *in silico* and *in vitro* safety assessments of pHLA complexes and related mismatched peptides is particularly useful when multiple TCR specific for the same pHLA ligand are available to be vetted against each other to select a final lead TCR, since the unique sequence of each candidate TCR will give it a unique pattern of cross-reactivity against mismatched peptides. TCR recognizing mismatched peptides that cannot be adequately de-risked through further studies can be abandoned and alternate TCR with better safer profiles given priority. In this way, the TCR with the best safety profile can be selected as the lead from a group of TCR sequences under comparison. Thereby, Expitope represents an innovation that plays an important role in both *Target Screening* and *TCR Discovery*.

### Tailored MS to validate pHLA complexes *in vitro*

The *Target Screening* module also includes an *in vitro* technology that allows peptides of a target antigen, which are predicted by Expitope to be presented by specified HLA allotypes, to be validated experimentally. For example, a target antigen can be explored for peptides that can bind to HLA-A allotypes encoded by HLA-A*01:01, HLA-A*02:01, HLA-A*03:01, or others. This analysis is done by transferring the HLA coding sequence of each single HLA allotype into the K562 cell line, which itself lacks endogenous HLA expression (77, 78). Thereafter, a target antigen is co-expressed in each K562 cell line. Surface HLA molecules are isolated and the mass spectra of eluted peptides are determined, as related to the target protein. We refer to this approach as *Tailored MS* since emphasis is placed on the identity of peptides derived from a selected target antigen that bind to a selected HLA allotype, in isolation from other HLA molecules. Similar MS approaches have been described previously (79, 80). In our approach, a tailored MS analysis, performed through an external service provider^9^ is complete when identified peptides have undergone an Expitope safety screen as described above to elucidate expression patterns in healthy tissues and to identify mismatched sequences for further studies of safety.

## Module 2: TCR generation

Once target antigens and HLA-peptide complexes have been identified using the *in silico* and *in vitro* technologies of Module 1, the next sequential step is to validate the individual predicted pHLA complexes for their potential to be recognized by T cells, leading to T cell activation. At the same time, T cell clones found to be capable of responding to any predicted pHLA ligand automatically become potential sources of TCR sequences that can be vetted for use in development of TCR-T therapies if they fulfill defined characteristics. Because pHLA complex identification is predicted on the basis of bioinformatics, patient materials in the form of TIL or peripheral blood T cells and tumor cells are not available for target antigen validation. As an alternative, *de novo* immune responses to predicted pHLA ligands can be validated using the T cells of healthy donors that are stimulated with antigen-presenting cells expressing the selected pHLA complexes identified *in silico* or by tailored MS *in vitro*. T cells responding *de novo* to predicted pHLA complexes can, in turn, be used to functionally validate the relevance of a selected target antigen in cancer cells by measuring T cell responses to panels of tumor cell lines identified to express the relevant antigen and HLA allotype on the basis of RNA and protein sequence information. In this way, the next sequential step in the E2E Platform is designed to deliver T cells and their corresponding TCR sequences independent of access to patient blood and/or tumor specimens and to simultaneously functionally validate the selected target antigens and pHLA ligands by TCR recognition.

**Figure 6:**
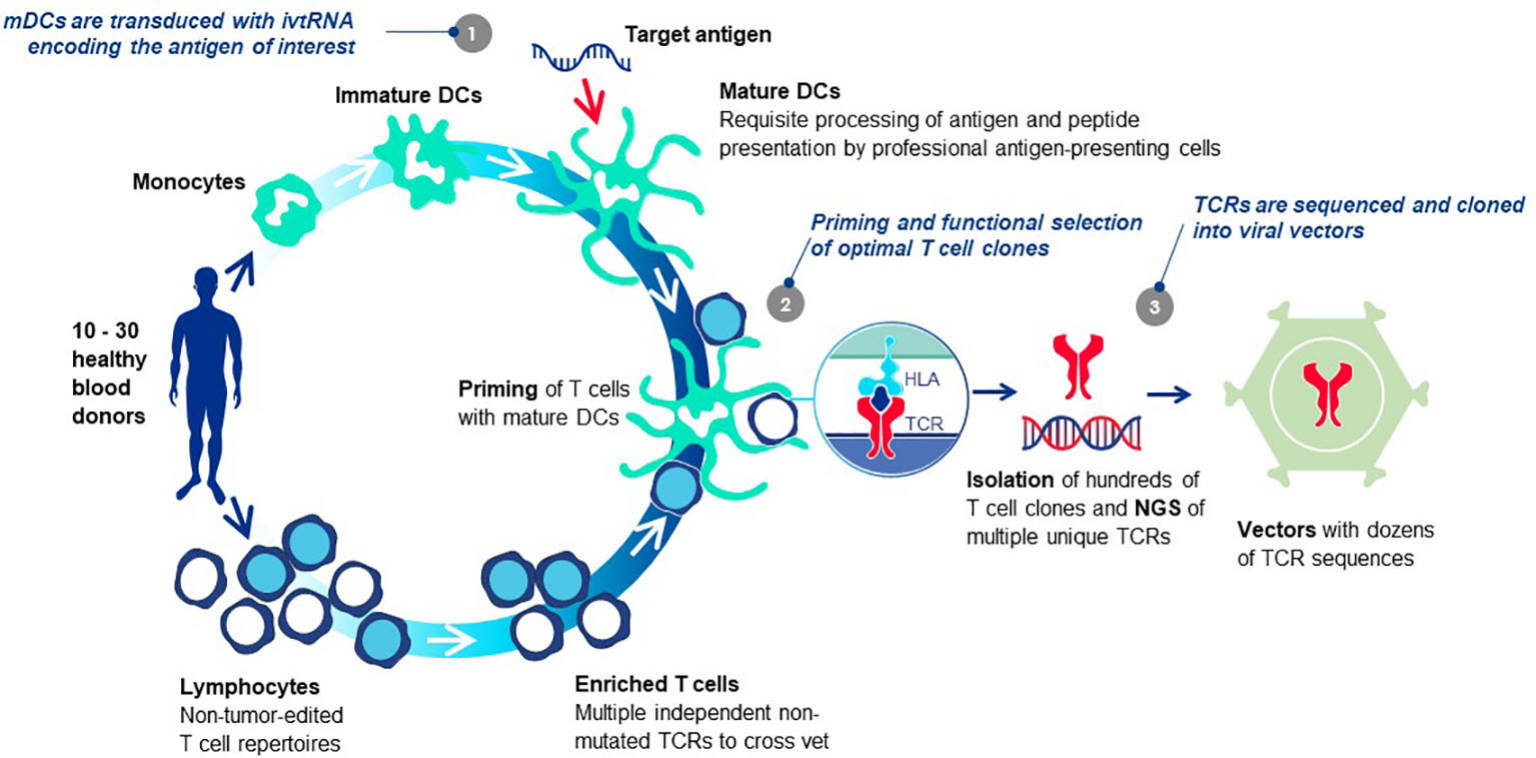
T cell-dendritic cell co-cultures prime antigen-specific T cells *de novo.* The T cells of healthy donors who have not been exposed to tumor antigens can be primed *de novo* using co-cultures of T cells and autologous mature DC *in vitro*, bypassing the need for patient blood or tumor specimens. The source of target antigen is *in vitro transcribed* RNA (ivtRNA), which is electroporated into the cytosol of the mDC and directly translated into protein. Thereby, pHLA complexes for T cell stimulation are generated by the natural intracellular machinery of the mDC and presented on the cell surface for stimulation of T cells with interacting TCR. In this way, antigenic peptides must compete for HLA presentation with peptides derived from other normal cellular proteins, limiting their expression to more physiological levels expected to be found on tumor cells. At the same time, a competition is set in place for T cells with TCR that have greater peptide sensitivity and can be activated by low levels of pHLA ligand expressed by the mDC, yielding TCR sequences of higher affinity in activated T cell clones. Use of multiple donors allows each priming cycle to yield TCR sequences that are unique for each donor. The diverse set of TCR, specific for the identical pHLA ligand, are vetted against each other after cloning into a retroviral vector and expression in transduced recipient T cells. Comparison of multiple rTCR with each other using a battery of functional assays allows the best sequence to be selected as the lead 3S TCR to be used for TCR-T therapy development, based on three characteristics: exquisite specificity, high sensitivity for target recognition and an excellent safety profile.

Each TCR selected for TCR-T therapy development must embody three essential properties: exquisite specificity, high sensitivity for tumor cell recognition and an excellent safety profile. We refer to these as “3S TCR” and an automated, high throughput screening (HTS) process is implemented in the *TCR Discovery* module to identify the rare TCR that meet our specifications. T cells of healthy donors are used to isolate T cell clones specific for pHLA complexes identified by the *Target Screening* module. Antigen-specific T cells are primed *in vitro* using autologous mature dendritic cells (DC) that can optimally prime naïve T cells (81) (**Figure 6**). Both CD4 and CD8 T cells can be primed if the mature DC are provided with target antigen for peptide presentation by class I HLA allotypes for CD8 T cells or class II HLA allotypes for CD4 T cells, respectively. Similar approaches are common to the field with different variations, some using DC and others based on alternate forms of antigen presentation to stimulate T cell responses. Most rely on peptide presentation by one of the self-HLA allotypes of the T cell donor, designated as autologous, or self-HLA priming (8).

After priming, antigen-specific T cells are selected by one of several known methods following antigen-specific stimulation (8) and sorted as single cell clones. With robotic support, tens of thousands of individual T cell clones can be isolated, expanded and functionally screened for pHLA specificity, normally based on antigen-induced cytokine secretion. TCR sequences of individual antigen-specific T cell clones are determined by NGS. Algorithms to match TCR alpha and beta chains are not required since the sequences are identified from expanded T cells originating from a single cell. Occasionally, two TCR alpha chain sequences will be identified and they must be compared in TCR-reconstituted cells to determine which chain is antigen-specific. When multiple donors are used, a collection of unique TCR can be assembled that have shared pHLA specificity but different sequences. TCR are expressed in recipient T cells using retrovirus-based gene transfer and direct comparisons of the different TCR are made using a battery of methods as described elsewhere (82, 83).

### Providing pHLA ligands for CD4 T cell priming with the Cross-TAg Vector System

**Figure 7:**
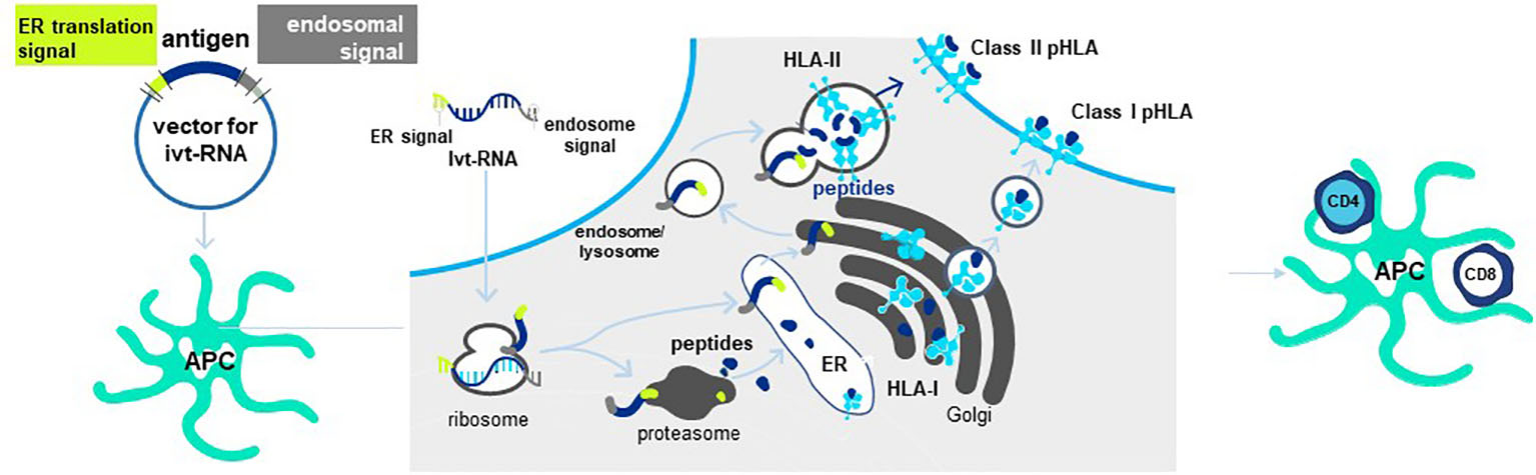
Cross-TAg Vector System targets peptides to both MHC class I and class II HLA molecules. Antigen processing and presentation for HLA class I and class II allotypes occurs in different intracellular pathways in mDCs. The Cross-Tag Vector System allows *ivt*RNA to be introduced into mDC via electroporation, which is directly translated into protein that contains different sorting signals at the N- and C-terminal ends of the protein. Sorting signals at both ends allow the protein to translocate to the endosomal compartment for antigen processing and presentation by HLA class II molecules for CD4^+^ T cell priming. Cytosolic protein enters the proteosome for processing and transport into the ER for binding to HLA and export and expression at the T cell surface for CD8^+^ T cell priming.

Antigen is provided to mature DC in the form of *in vitro* transcribed RNA (ivtRNA) that is directly introduced into the cytosol by electroporation, where it is rapidly translated into protein (84). In this way, the intracellular machinery of the DC used for antigen processing and presentation creates pHLA complexes from the provided antigen and class I HLA allotypes to stimulate purified CD8 T cells. To prime purified CD4 T cells, a change is made in the ivtRNA since a different intracellular pathway must be engaged to create HLA class II peptide complexes (8). This occurs in an endosomal-lysosomal compartment in DC to which cytosolic proteins only gain entry if they contain specific sorting signals. The proprietary *Cross-TAg Vector System* (**Figure 7**) adds sorting signals to a target protein as it is translated from the ivtRNA template in the cytosol of the DC. These sorting signals enable the protein to translocate to the endosomal-lysosomal pathway where peptides can be processed and selected by binding to class II HLA allotypes for transport to the cell surface, where they can activate CD4 T cells with complementary TCR (85–89).

### Allo-HLA TCR Priming to acquire natural high-affinity TCR

*Allo-HLA TCR Priming* represents a proprietary specialized innovation designed to acquire natural higher-affinity TCR recognizing peptides derived from self-proteins, without need to artificially adjust or mutate the TCR sequence (referred to as affinity maturation) to improve sensitivity (8, 90). Natural high-affinity TCR can be directly isolated by introducing a simple change in the priming procedure described above. A non-self-HLA allotype is added to autologous DC and used for HLA allo-restricted peptide presentation and stimulation of autologous donor T cells (82, 83, 91, 92). The original precedent for this strategy was shown for allo-restricted T cell priming by an allogeneic antigen-presenting tumor cell line pulsed with exogenous peptide (93).

**Figure 8:**
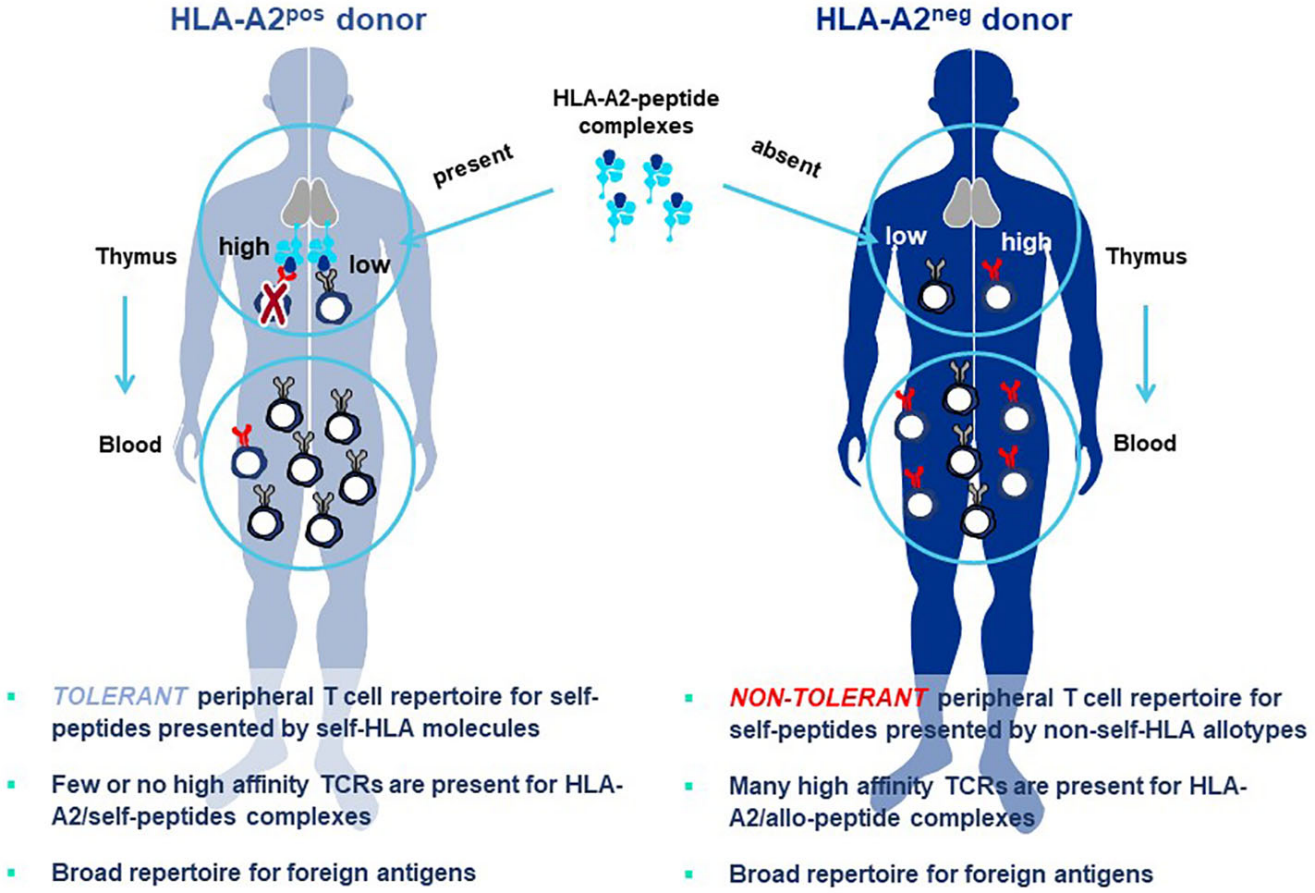
Principle of Allo-HLA TCR Priming to bypass central tolerance. T cells with high-affinity TCR capable of binding self-peptides presented by autologous HLA molecules in the thymus are deleted through the mechanism of central tolerance to protect against autoimmunity (left figure). The resultant tolerant peripheral T cell repertoire circulating in blood and available for study is comprised of T cells with only low-affinity TCR that can recognize self-peptides, with high-affinity TCR being a very rare exception. This not the case for peripheral T cells of a donor who does not carry the HLA allele encoding the HLA allotype used for self-peptide presentation (right figure). Here T cells with high-affinity TCR do not encounter the self-peptide/allo-HLA ligand in the thymus. Thus, the circulating T cell repertoire is comprised of a non-tolerant T cell repertoire for the allo-HLA that has TCR that use natural non-mutated sequences to recognize self-peptides with high-affinity. The difference in outcomes on TCR affinity in the two donors is not related to the pHLA complex recognized by the TCR, since these are identical in both cases; rather the differences in TCR affinity are related to the status of tolerance of the responding T cell repertoires from which the TCR are derived. Both donors have peripheral T cell repertoires with high-affinity TCR that recognize foreign peptides, since such pHLA complexes are not presented to T cells in the thymus of either donor where they would be subjected to deletional tolerance.

The principle underlying Allo-HLA TCR Priming is illustrated in **Figure 8**. The T cell repertoire of a normal donor is depleted of T cells with high-affinity TCR specific for self-proteins by a mechanism of T cell deletion in the thymus, known as central tolerance. T cells with higher-affinity TCR specific for self-peptide/self-HLA complexes are eliminated to prevent autoimmunity (94) and T cells exiting the thymus and circulating in peripheral blood will have only lower-affinity TCR for self-peptides. When T cells are primed with DC that co-express a self-protein antigen, such as a CGA, and an additional HLA allotype not present in the T cell donor (i.e. non-self-HLA), higher-affinity TCR can be isolated since responding T cells have not undergone deletional tolerance in the thymus. As examples, an HLA-A2-positive donor has mostly lower-affinity T cells capable of recognizing self-peptide/HLA-A2 complexes due to deletion of higher-affinity TCR in the thymus, with an occasional exception (**Figure 8** left). In contrast, an HLA-A2-negative donor has a peripheral T cell repertoire with many higher-affinity TCR that recognize identical pHLA-A2 complexes, but with greater sensitivity since deletional tolerance has not removed higher-affinity TCR that recognize the same self-peptides presented by HLA-A2 molecules (**Figure 8** right). Here the difference in outcome on TCR affinity is not related to the pHLA complex recognized by the TCR, but rather to the status of tolerance of the responding T cell repertoire from which the TCR are derived.

TCR are comprised of an alpha and a beta chain that form a TCR heterodimer which directly engages a complementary pHLA ligand. Upon sufficient interaction, TCR-associated CD3 molecules transduce signals that activate intracellular pathways governing T cell response. Both TCR chains have variable and constant region domains. The two variable region domains determine TCR specificity for the complementary pHLA target ligand. Variable regions of TCR are highly diversified so that each naïve T cell will express a unique TCR sequence. The strength and duration of TCR-target cell interactions determine whether a T cell will respond and the type of T cell response that will develop (8, 91, 92, 95). If a TCR is isolated using autologous-HLA priming which is specific for a self-peptide, modifying the TCR sequence is likely to be needed to improve pHLA sensitivity and determine the function of TCR-T cells. Hereby, mutations are introduced in the variable regions of the TCR to improve contacts with the pHLA complex, in a process known as *affinity maturation* (8). These mutations must be carefully vetted to assure that specificity and safety of the mutated TCR is not altered (29, 75).

In order to increase the affinity of a TCR through mutation, one normally starts with a single TCR sequence from which a large number of mutated TCR variants are generated and screened to select a TCR sequence that has higher affinity for the pHLA ligand but still has an acceptable safety profile (8, 90). The resulting mutant TCR variants represent highly related sequences, often differing only by single amino acid exchanges that still permit pHLA specificity to be retained. Allo-HLA TCR Priming obviates the need to mutate TCR sequences to obtain higher-affinity TCR. It also brings an additional advantage with respect to TCR sequence diversity, since each TCR isolated from a different healthy donor has a unique, fully unrelated TCR sequence that differs from those of other healthy donors in multiple amino acids that interact with the pHLA ligand. Thus, TCR isolated from different donors provide far more diversity than mutated TCR variants from which to identify optimal 3S TCR.

Furthermore, the non-mutated TCR are isolated from natural repertoires of T cells that are circulating in healthy donors without signs of autoimmunity, giving them an additional safety dimension. It should be noted that isolation of TCR that recognize foreign peptides, such as those from viral proteins, MHA, or mutated proteins, can rely on the more simple approach of self-HLA priming (**Figure 8** left), since deletional tolerance against foreign antigens does not occur in the thymus. Thereby, TCR specific for foreign pHLA complexes can be found with natural higher-affinity that do not require mutation to show sensitive recognition of their pHLA ligands.

The inclusion of many healthy donors in Allo-HLA TCR Priming experiments is designed to acquire multiple unrelated TCR sequences to vet against each other to find the lead candidate that embodies the properties needed to qualify as a 3S TCR. Although every TCR is initially selected to recognize wild-type pHLA ligand, different TCR will display subtle differences in recognition of peptide presented by closely related HLA allotypes, which is designated as HLA fine specificity. For example, some TCR can recognize peptide presented by highly related HLA-A*02:01- and HLA-A*02.06-encoded HLA allotypes, but others not. The TCR will also vary in peptide sensitivity and their ability to recognize tumor cells with lower levels of antigen. Safety profiles of individual TCR will be distinct, responding to different mismatched peptides due to their unique TCR sequences. Some TCR may bind to unrelated HLA allotypes, which is designated as HLA allorecognition, but others will not show this property. By isolating groups of TCR with identical pHLA specificity through Allo-HLA priming and HTS, diverse TCR are obtained and extensively compared with each other to select the best 3S TCR for TCR-T therapy development.

**Figure 9:**
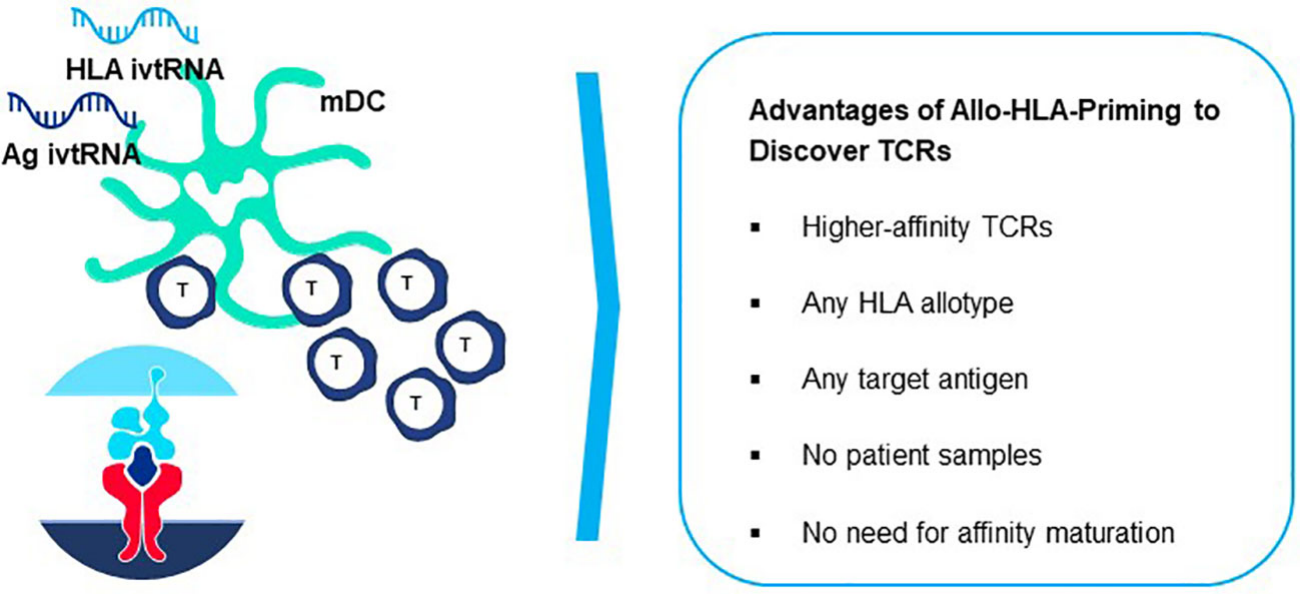
Generation of peptide-allo-HLA complexes on mature dendritic cells for *de novo* T cell priming. Based on the principle of tapping non-tolerant T cell repertoires to isolate natural high-affinity TCR, T cell-mDC co-cultures can be used to isolate highly sensitive TCR by preparing mDC that are electroporated simultaneously with *ivt*RNA encoding the target self-antigen and *ivt*RNA encoding the allo-HLA molecule used for self-peptide presentation. New allo-HLA self-peptide complexes are displayed on the surface of the mDC, generated from the two supplied *ivt*RNAs and these allo-pHLA ligands serve to stimulate non-tolerant autologous T cells with high-affinity TCR.

In general, TCR with higher peptide sensitivities are activated through Allo-HLA-Priming (82, 91, 92). These TCR also trigger secretion of cytokines that are Th1-like in composition which contribute to effective antitumor responses, whereas self-peptide/self-HLA primed T cells often show patterns more like Th2 cells that are less well suited for responses against tumor cells (92, 93). The Allo-HLA TCR Priming approach has great versatility since it can be used with any HLA allotype simply by using T cells of donors who lack the HLA allotype used for peptide presentation (**Figure 9**). This highly flexible approach allows higher-affinity TCR seeing target peptides presented by different HLA allotypes to be directly generated for treatment needs of patients with diverse HLA alleles. Furthermore, higher-affinity TCR incorporated in TCR-T therapies will have improved capacity to combat cancers with lower target antigen levels. Nevertheless, a delicate balance much be reached between improved cancer recognition and potential cross-reactivity that can occur with higher-affinity TCR. Allo-HLA TCR Priming of multiple donors provides diverse TCR sequences for these comparisons and improves the chances that a natural TCR sequence can be found with an *optimal affinity* that is broadly sensitive for tumor cell recognition but retains an excellent safety profile.

The combined use of Allo-HLA TCR Priming and HTS robotics is a technological approach that goes far beyond the selection of a lead TCR for a given target antigen. The expansion of TCR-T therapy to patients with diverse HLA backgrounds can be met through use of diverse peptides derived from the same target antigen with different HLA allotypes, or through use of different antigens to form pHLA ligands suited for treatment of the same indications. On one hand, this increases the numbers of patients who can benefit from TCR-T therapies but it also opens the door to combine TCR-T cells of different pHLA specificity to combat tumor cell heterogeneity. Mixtures of TCR can continue to recognize and destroy tumor cells that differentially lose expression of a single antigen or HLA allotype (96–98). Allo-HLA TCR Priming has been successfully applied to isolate TCR recognizing more than a dozen different targets, drawn from the six major categories of antigen considered relevant for TCR-T cells (**Figure 3**), including differentiation antigens (91), universal antigens (38, 91), viral antigens (99), CGA/CTA (82, 83), as well as MHA and neoantigens^10^ ^,^ ^11^. The approach has also been applied for multiple HLA allotypes, including several of those shown in **Figure 5** that represent common HLA alleles^12^ found worldwide. These findings demonstrate the great flexibility that can be used to isolate TCR to build a compelling TCR-T therapy pipeline of the future, with broad expanse to meet the needs of patients worldwide with many types of cancer.

### Jovi-Tag to track and enrich TCR-T cells expressing recombinant TCR

**Figure 10:**
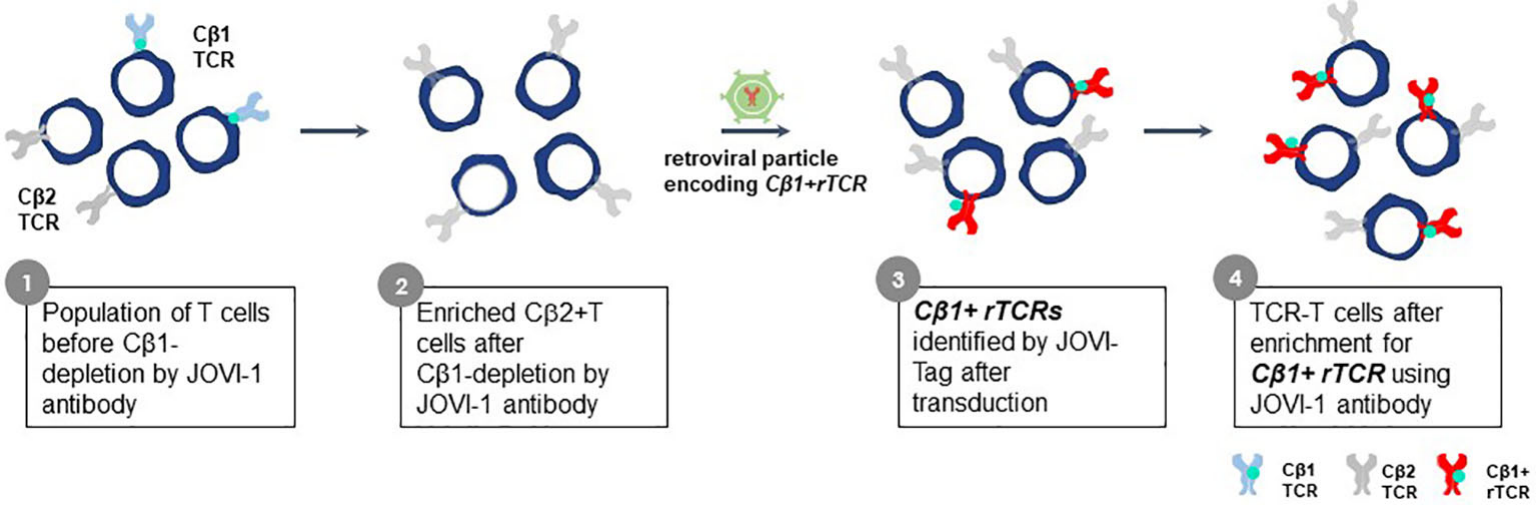
Jovi-Tag as a TCR marker to track and enrich TCR-T cells with rTCRs. TCR beta chains of individual TCR use constant region domains encoded by TRBC1 or TRBC2 gene sequences. The TRBC1 constant region domain displays an epitope that can be detected by the Jovi-1 antibody. Approximately equal numbers of peripheral blood T cells in healthy donors will express TCR using the TRBC1 or TRBC2 constant regions. The Jovi-1 antibody can be used to remove the T cells with TRBC1 constant regions (about 50%), leaving behind a purified population of T cells expressing TRBC2 constant region domains that do not bind Jovi-1 antibody. If rTCR are cloned to use TRCB1 constant regions, upon introduction into TRBC2 recipient T cells, they can be identified by their ability to bind Jovi-1 antibody, irrespective of their variable regions. This allows expression levels of rTCR to be determined and homogenous populations of TCR-T cells expressing rTCR can be selected through Jovi-1 antibody binding and sorting by flow cytometry or through magnetic enrichment technologies.

The constant region domains of a TCR contribute to TCR heterodimer pairing and govern interactions with CD3 proteins. Alpha chain constant region domains are monomorphic in all TCR whereas TCR beta chains in individual TCR use one of two different constant region domains, encoded by TRBC1 or TRBC2 gene sequences (100). The Jovi-Tag uses a sequence variation that distinguishes TRBC1 and TRBC2 constant region domains to provide an innovative method to accurately compare different rTCR for lead TCR selection, independent of pHLA specificity. This proprietary technology allows standardized comparisons of different rTCR for surface expression and function. The beta chain of every rTCR is constructed with a TRBC1 constant domain that binds the Jovi-1-specific antibody (101) (**Figure 10**). CB1-positive rTCR are thereby uniquely tagged if they are expressed in purified CB2-positive recipient T cells, which themselves do not bind Jovi-1 antibody. All rTCR are easily assessed for variations in surface expression by their Jovi-Tag and enriched populations of TCR-T cells can be isolated with the Jovi-1 antibody to provide homogenous T cell populations for functional studies of specificity, sensitivity and safety. Murine constant region domains have been used to reconstruct human rTCR sequences, for which a specific detection antibody is also available (102). However, use of human CB1 domains in rTCR brings the major advantage that they comprise only human sequences found in natural TCR and avoid impacts on 3S TCR characteristics that could be subtly altered using mixed species domains. Inclusion of murine constant region domains in final TCR-T therapies also carries some risk for immunogenicity if they are used *in vivo*.

## Module 3: TCR-T therapy optimization

Once a 3S TCR has been selected that shows effective tumor recognition, further considerations are made with respect to how such a TCR can be enhanced through innovation to utilize it in an optimal TCR-T therapy. This requires consideration of the properties of recipient T cells that determine how effective TCR-T therapies are at gaining control and, in the best case, eradicating cancer in a patient. Innovations to optimize TCR-T therapies covered in the third module of the E2E Platform are made at two levels: rTCR structures are directly altered to change their expression in recipient T cells and recipient T cells are engineered to improve their functional attributes to better combat tumor cells. These innovations can be combined to further enhance TCR-T therapies.

A 3S TCR is extensively vetted for antigen sensitivity and safety during lead TCR selection, but additional considerations come into play upon introduction of a rTCR into recipient T cells, which impinge on both function and safety. Every recipient T cell expresses a different endogenous TCR and some endogenous chains may form heterodimers with rTCR chains and interfere with optimal rTCR surface expression. There are strong structural constraints on formation of mixed TCR heterodimers by the variable regions of the TCR alpha and beta chains that must intimately fold with each other (103, 104); nevertheless TCR mispairing does occur. Formation of TCR mixed heterodimers decreases rTCR surface expression by limiting the numbers of rTCR chains needed for correct pairing with each other, if mispairing is extensive. In addition, expression of mixed TCR diminishes the amount of CD3 available for association with correctly paired rTCR heterodimers. Fewer surface rTCR lowers the functional avidity of TCR-T cells that depends on TCR affinity and levels of TCR surface expression (8). Lower functional avidity, in turn, is highly detrimental for TCR-T recognition of tumor cells with low amounts of pHLA ligand.

Mixed TCR heterodimers can also create safety concerns as some mixed TCR theoretically could recognize healthy cells through display of new specificities, detracting from the safety profile of TCR-T cells. Endogenous TCR chains that formed mixed heterodimers with rTCR chains were reported to cause lethal toxicity in a mouse model (105). However, such toxicities have not been reported in clinical TCR-T trials to date (7–9). Fortunately, measures to mitigate TCR mispairing are available (7–9), with knock-out of endogenous TCR genes representing the most recent and sophisticated approach (7, 9, 42). This method gains traction as gene editing technologies improve. It is still a major challenge, however, to use for patient-individualized TCR-T therapies since both TCR genes must be incapacitated in every cell to totally eliminate mispairing of both endogenous TCR chains with the reciprocal chains of the rTCR. This is only now being developed for successful implementation in TCR-T manufacturing (42).

### Improvement of TCR-T functional avidity and safety through Precision Pairing and Inducible TCR

**Figure 11:**
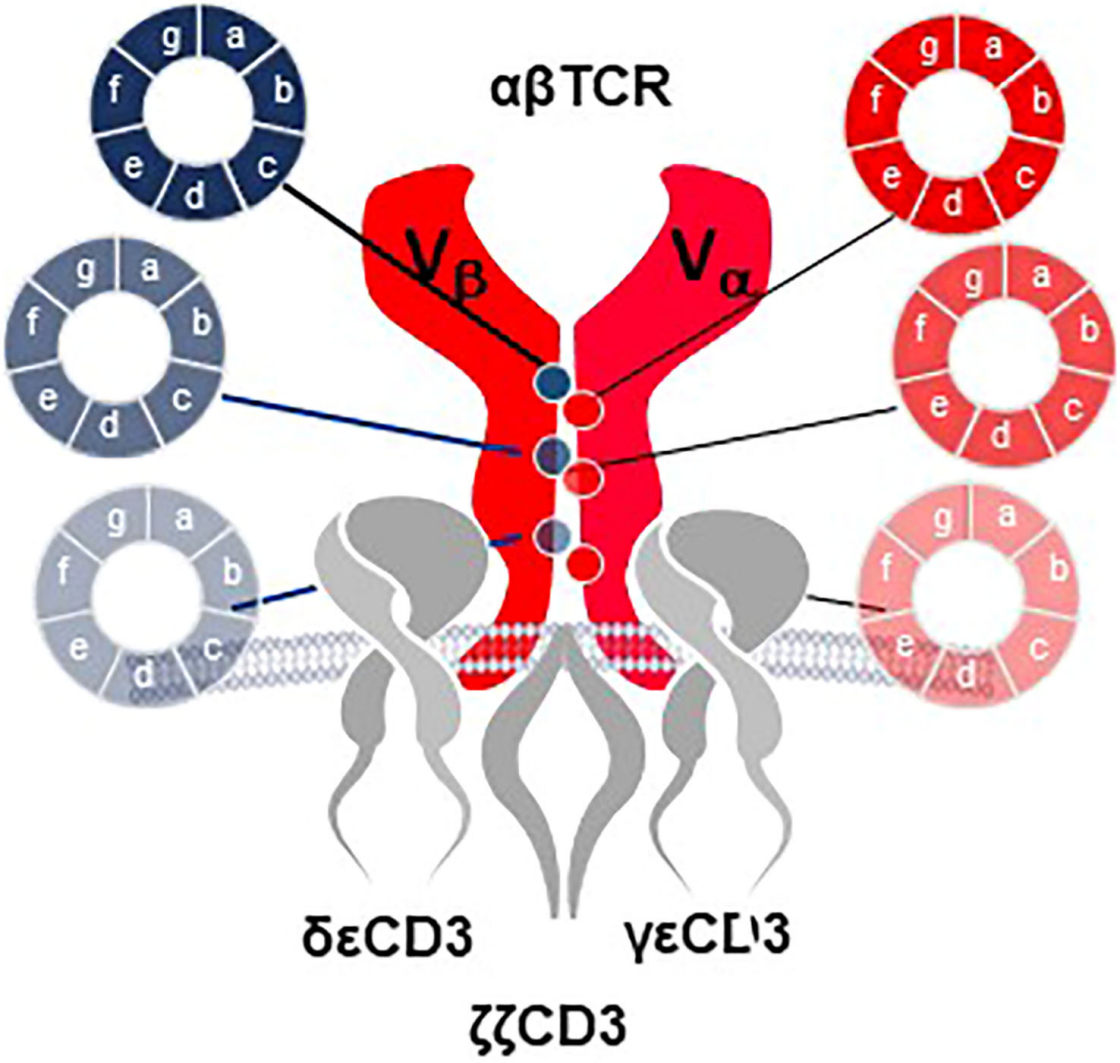
Precision Pairing tailors TCR heterodimers for optimal interaction. A complex library of TCR constant region domain sequences is developed for combination with the variable regions for each TCR chain. The constant region sequence variations encode various amino acids in selected positions that contribute to TCR heterodimer pairing and interaction with the CD3 complex. Selection of TCR chains that utilize constant region variants that lead to improved interactions allow the TCR alpha and beta chains to be tailored for precision pairing. Selection of precision-paired TCR is done in sequential enrichment steps that ultimately allow the amino acid sequences to be identified by sequencing of TCR that show improved surface expression compared to wild-type constant region domains or for TCR that display improved functional capacity for titrated antigen recognition based on improved T cell functional avidity through increased TCR surface expression.

Two innovations in the E2E Platform introduce structural changes in rTCR to improve function and safety of TCR-T cells. *Precision Pairing* is a proprietary technology that tailors both chains of a rTCR to have unique constant region domains that precisely guide rTCR pairing, without interfering in the specificity of their variable regions. This is accomplished by selecting precision-paired constant region domains from an extensive library of constant region sequences that vary in amino acids at defined positions that are involved in TCR chain pairing (**Figure 11**). Precision-paired rTCR show higher surface expression leading to enhanced functional avidity^13^.

**Figure 12:**
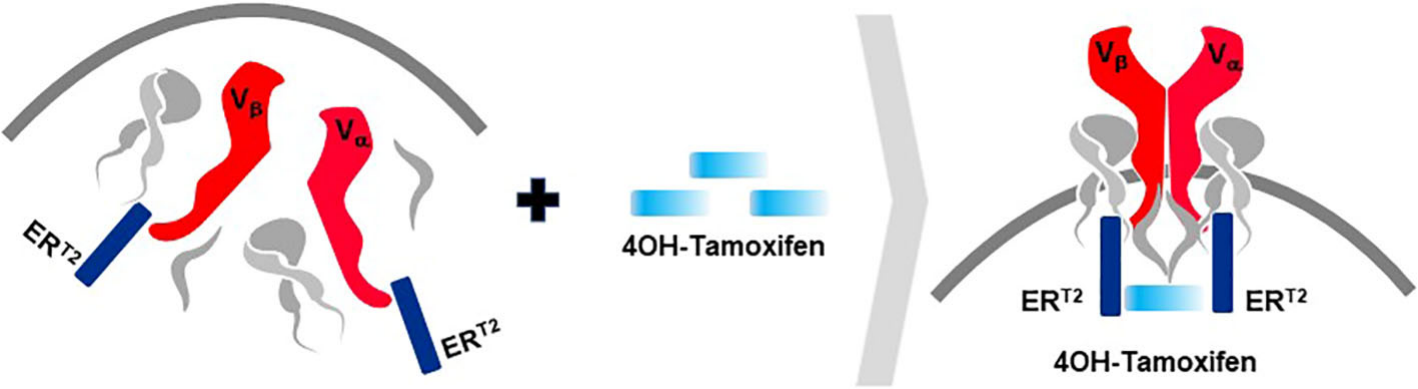
Inducible iM-TCR System provides exquisite control of TCR heterodimer formation. Strict control of rTCR surface expression can be obtained with the inducible iM-TCR System that only allows TCR heterdimers to be formed and associate with the CD3 complex in the cytosol of TCR-T cells upon induction using 4OH-Tamoxifen. The TCR heterodimers (red structures, upper and lower figures) contain mutated constant region domains that do not allow them to pair with each other or with endogenous TCR chains in recipient T cells (not shown). Individual domains of the estrogen receptor (ER^T2^) are added to the ends of the two TCR chains (dark blue structures, upper and lower figures). In the absence of TCR heterodimer formation, the components of the CD3 complex and the zeta chains (grey structures, upper figure) remain unassociated with the rTCR chains in the TCR-T cytosolic compartment. Upon addition of 4OH-Tamoxifen (light blue bar), TCR heterodimers are formed, CD3 molecules and zeta chains associate with the paired TCR chains and the TCR-CD3 complex moves to the surface of the TCR-T cells where it can function as a normal TCR-CD3 complex that can recognize antigen and activate intracellular signaling in response (lower figure).

A second rTCR structural change is embodied in the proprietary *iM-TCR System* that improves both efficacy and safety of TCR-T therapies in a highly regulated fashion^14^. The iM-TCR System is designed for strict control of rTCR surface expression on TCR-T cells while it completely abolishes mispairing of recombinant and endogenous TCR receptor chains, without need to knock-out endogenous TCR. Each chain of the iM-TCR is mutated in the constant region so it cannot engage in any TCR heterodimer formation. Each chain is then engineered to express a mutated estrogen receptor domain (ER^T2^) (106, 107), attached to its intracellular tail (**Figure 12**). Upon addition of 4OH-Tamoxifen or Endoxifen, the ER^T2^ domains undergo intracellular dimerization that allows pre-existing unpaired alpha and beta chains to form rTCR heterodimers and move to the TCR-T cell surface in a time- and concentration-dependent manner. Induced iM-TCR show increased surface expression and improved functional avidity upon induction^15^.

Additional characteristics of iM-TCR contribute to the unique attributes of this innovation, based on the ability of iM-TCR to be toggled between the cell surface and the cytosol of TCR-T cells, via ligand-induced control of TCR heterodimer formation (**Figure 12**). Turnover of TCR is a highly regulated natural process and maintenance of surface TCR expression depends on resupply by cytosolic TCR heterodimers that combine with CD3 for transport and expression at the cell surface (108–110). Since iM-TCR resupply is fully dependent on controlled cytosolic dimerization of iM-TCR heterodimers, a relatively rapid loss of rTCR from the cell surface in the absence of iM-TCR resupply can be used as a safety control to tame unwanted TCR-T cell activities. In another use, pausing dimerization temporarily stops iM-TCR resupply to the cell surface and allows TCR-T cells to rest and recuperate from exhaustive TCR signaling that occurs in a solid tumor. After this period of rest, iM-TCR expression can be restored and TCR-T cells will regain their potent effector functions. Similar approaches have been devised to control CAR-T expression (111). Regulated TCR expression has also been explored at the level of TCR gene transcription and translation (8), although these forms of regulation require more time to manifest changes in TCR surface expression, dependent on the half-life of TCR RNA or protein.

The ability to exquisitely control rTCR surface expression and rapidly tame TCR-dependent cellular functions, without eradicating the TCR-T cells, is a major attribute of the iM-TCR System. Also controlling duration of rTCR expression provides a mechanism to retain TCR-T cell persistence in situations of chronic stimulation, as found in a TME, potentially allowing TCR-T therapies to provide clinical benefit to patients for longer periods of time. It is also feasible to induce iM-TCR surface expression *ex vivo* and create TCR-T cells that interact with their target ligands for a short, well-specified period of time and then lose capacity to react to pHLA ligand if new TCR-T cells are not applied or TCR surface expression is not resupplied by provision of 4OH-Tamoxifen *in vivo*. In this way, highly controlled TCR-T therapies can be considered for treatment of cancer indications such as brain tumors, where local inflammation from continual TCR-T cell activity can be highly detrimental.

### Impacts of the TME on trafficking and function of T cells and other immune cells

Trafficking of immune cells is complex and impacted by multiple factors that impede T cell infiltration into tumors. The most prominent limitations of cell entry into a TME, including T cells and other immune cells, include physical barriers created by stromal cells, such as fibroblasts, that block penetration; abnormal vasculature that disturbs entry through the endothelium; and presence of immunosuppressive cells that disturb generation of lymphoid-like structures that attract diverse cells to an inflamed tissue through soluble signals (112, 113). Chemokines and chemokine receptors displayed by various cell types shape the TME with different receptor pairs supporting or suppressing TME recruitment of distinct cell types (113). The cellular and molecular composition of the TME also varies by indication, with some tumors better understood than others (113). Successful trafficking of T cells and function in the TME is nicely evidenced by abundant TIL found in melanomas (3, 4). These tumors have long served as models to dissect mechanisms that contribute to T cell trafficking.

One of the first obstacles met by T cells moving to gain access is a stromal barrier that inhibits cell penetration in many tumors. This physical barrier can be breached if T cells are able to secrete interferon-gamma (IFNγ) and tumor necrosis factor alpha (TNFα) (114, 115), two factors that are prominently displayed by 3S TCR. Secretion is dependent on TCR signaling in the T cells through stromal cell presentation of the target pHLA complex, which can occur by cross-presentation of antigen acquired from dying tumor cells and even increased through some types of chemotherapy (116, 117).

These two cytokines also play a role in altering the tumor vasculature to enhance leukocyte infiltration through the endothelium (118–120). There is good evidence that CD40 expression by tumor endothelium is engaged by CD40L^+^ T cells that secrete IFNγ and TNFα, leading to expression of adhesion molecules, like intracellular adhesion molecule-1 (ICAM-1), E-selectin (CD62E) and vascular cell adhesion molecule-1 (VCAM-1), which are required for trafficking and trans-endothelial migration (121). Activated endothelial cells produce additional proinflammatory cytokines that further enhance cell entry into the TME. Some extravasating cells will take up residence in lymph node-like structures that provide chemokine and interleukin signals to sustain immune cell trafficking and intra-tumor function (122, 123). Expression of CD40L is primarily associated with CD4 T cells but there is also a fraction of CD8 memory T cells that express CD40L and impact this process (124, 125).

Diverse factors including soluble mediators, surface molecules and costimulatory pathways all play a role in determining the types of cells that traffic to the TME, their numbers and their functions at tumor sites (113, 121). Specific chemokine–chemokine receptor signaling pathways control recruitment of CD8 T cells, CD4 T helper 1 (Th1) cells, or NK cells, among others. Likewise, DC are recruited to function as APC to present antigen and expand T cells with new specificities as well as to secrete cytokines to recruit and diversify antitumor immunity. As chemokines are central for T cell trafficking, one approach to enhance infiltration into the TME is to express relevant chemokine receptors in TCR-T cells or imbue them with capacity to secrete interleukins, like IL12, that activate APC to recruit further immune cells and broaden antitumor immunity (113, 126). However, given the substantial redundancy in chemokine-chemokine receptor interactions, it may be difficult to achieve meaningful clinical responses using single chemokine components, based on experience treating inflammatory diseases (113). Therefore, more complex interventions are likely needed to successfully influence trafficking of T cells and other immune cells to the TME.

**Figure 13:**
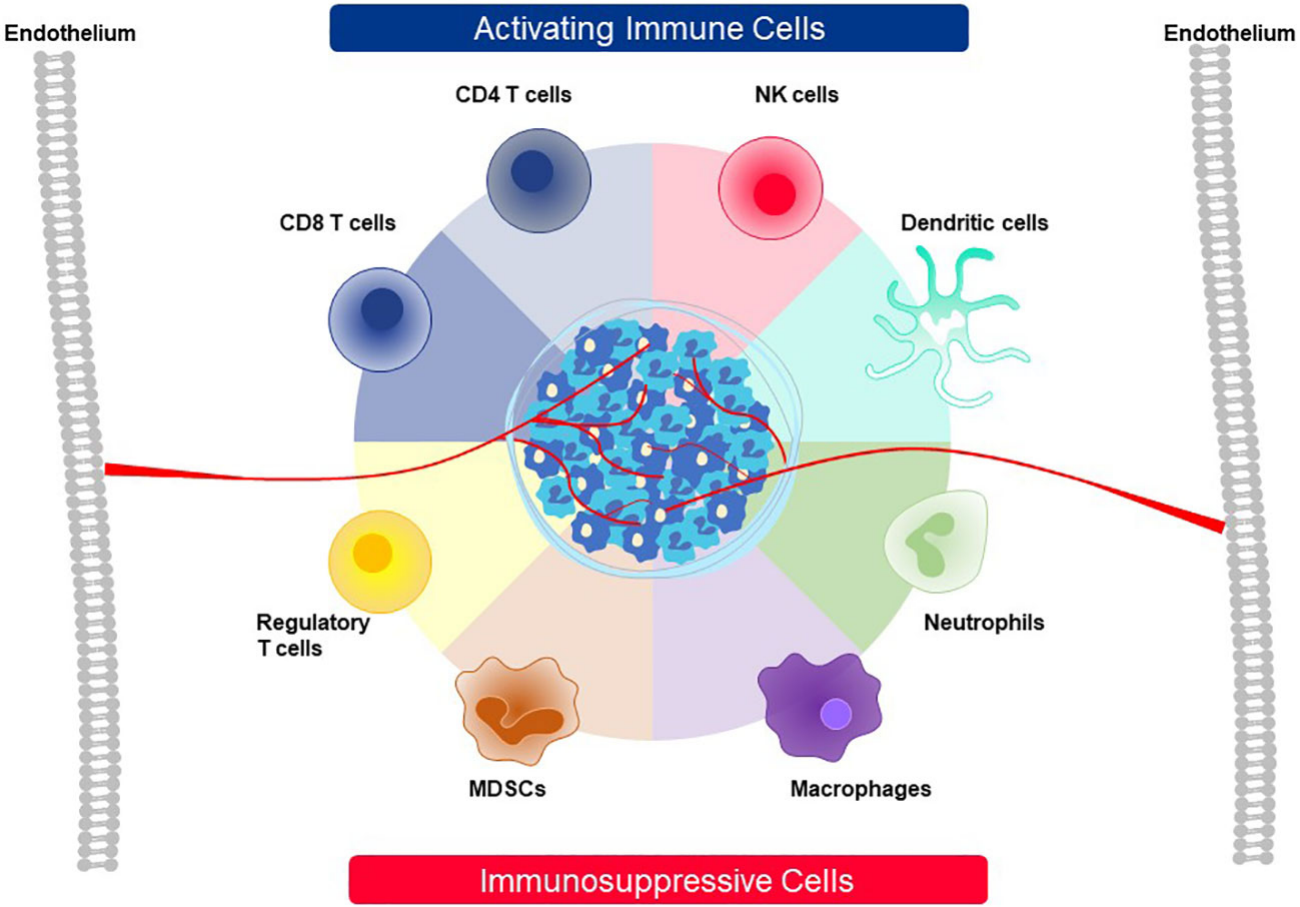
Cellular players contributing to activation or suppression of antitumor immunity in the TME. The TME of solid cancer is comprised of tumor cells and stromal cells, like fibroblasts, that build a barrier around many tumor masses. A variety of cell types that function either as activating cells that contribute to active antitumor immunity or, alternatively, are involved in immunosuppression of immune responses against cancer can be recruited to the TME. The roles played by the eight cell types depicted here are described in detail in the text.

A mixed composition of immune cells at a tumor site initiates competition between tumor cells and other accessory cells that work to benefit the tumor versus immune cells that must shape the TME and eliminate tumor cells (**Figure 13**). Tumor cells and cancer-associated fibroblasts form the basic cellular structure in which other accessory cells accumulate and which immune cells must overcome. Accessory cells recruited by tumors provide factors needed to support tumor growth and suppress immune responses. Prominent examples are tumor-associated macrophages (TAM), tumor-associated neutrophils (TAN), mesenchymal-derived suppressor cells (MSDC) and regulatory T cells (Treg) (7–9). On the side of anti-tumor activity, CD4, CD8, and NK cells as well as DC can build effective immune responses against a tumor (7–9). After CD8 T cells enter the TME through the tumor vasculature, live cell imaging has revealed that they accumulate at vessel sites where they cross into the TME and initiate cytotoxic attack against nearby tumor cells (127). In a slow and systematic manner the CD8 T cells then extend their killing field to larger regions as they move deeper into the tumor interior. If they display particular attributes, as described below, they can interact with some accessory cells and convert their immunosuppressive responses into antitumor functions. For example, CD4 T cells can employ several mechanisms to participate in tumor attack. Through secretion of IFNγ, they enhance presentation of pHLA complexes on tumor cells and directly support CD8 T cells. Some CD4 T cells can directly kill tumor cells. If the target antigen is transferred and cross-presented by endothelial cells, they can also destroy the tumor vasculature (128, 129).

Because CD4 T cells see their target ligands naturally through presentation by HLA class II molecules, they can only naturally recognize tumor cells that are HLA class II positive. To date, most TCR used in TCR-T therapies are specific for class I pHLA ligands. This creates a difficult situation for use of CD4 T cells in addition to CD8 T cells in ACT since patients need to be selected on the basis of HLA class I and class II allotypes matching the restriction specificities of the CD8 and CD4 T cells, respectively, even if they recognize peptides derived from the same target antigens (128). This could dramatically limit the numbers of available patients. It is far more feasible to express one HLA class I-restricted TCR in both CD4 T and CD8 T cells. This can occur if TCR are selected that are CD8 coreceptor independent (7, 8). Our proprietary screening algorithm searches for such TCR while vetting multiple candidate sequences during lead 3S TCR selection (82). Alternatively, CD8 molecules can be expressed as transgenes in CD4 T cells to enable proper signaling of class I restricted TCR (7, 30), however this approach requires more complex vector engineering to accommodate additional genes encoding CD8 alpha and beta chains and ensuring their stable expression in TCR-T cells.

### Designing TCR-T cells to overcome negative effects of the TME

If TCR-T cells successfully gain entry into the TME there are many additional facets that govern whether they will mount highly effective antitumor responses and achieve eradication of heterogenous tumor cells in a cancer mass. One of the most critical properties of TCR-T cells is the capacity to expand to large numbers by rapid proliferation to effectively combat large tumor burdens. At the same time, some TCR-T cells must persist as long-term memory cells and retain their capacity to re-initiate expansion after renewed antigen-specific stimulation, a characteristic described as stemness (8, 130–132). TCR-T cells must embody a plethora of effector mechanisms to directly control tumor cell survival and also to participate in recruitment of additional cells to an antitumor response. In an optimal scenario, production of diverse cytokines and cytotoxins by TCR-T cells upon TCR binding to pHLA ligands imbues them with capacity to directly attack tumor cells, primarily mediated by CD8 cytotoxic T cells that express high levels of cytotoxins (8, 133–135). At the same time, TCR-T cells should initiate a cascade of subsequent events that alter the TME and enhance immune responses that are dependent on poly-cytokine responses by TCR-T cells.

Recognition and killing of tumor cells leads to release of antigen mixtures by dying tumor cells that can, in turn, activate T cells with new antigen specificities and expand the repertoire of effector T cells that contribute to fulminant antitumor responses, in a process known as *antigen spreading* (136–140). Expansion of an antitumor T cell repertoire is important to eliminate tumor cells that no longer express the pHLA complex that drives the initial TCR-T cell response. TCR-T cells can also initiate recruitment of innate immune cells, like NK cells, that attack tumor cells which no longer express HLA (8, 9). Both of these activities are fostered by TCR-T cell secretion of multiple cytokines, particularly IFNγ. TCR-T cells with capacity to produce complex mixtures of cytokines can stimulate antigen-presenting cells, including DC, which are present but may not be active in the TME. Activated antigen-presenting cells further amplify immune attack through recruitment of new effector cells. All of these mechanisms are most pronounced when TCR-T cells are able to sustain killing and poly-cytokine secretion (8), despite persistent exposure to tumors cells and in the presence of immunosuppressive mechanisms mobilized by tumor cells to block effective TCR-T cell responses.

New approaches are being deployed to armor TCR-T cells to avoid immunosuppressive signals, such as those driven through the PD-1/PD-L1 axis, Fas/Fas ligand (FasL) interactions, and tumor growth factor beta (TGFβ) receptors, to name some examples (7–9). These negative signals to TCR-T cells can be countered using gene knockout technologies to remove surface expression of relevant receptors or by using dominant negative receptors to reduce the negative signals in TCR-T cells (8, 9). At the same time, TCR-T cells can be engineered to enhance their capacities to proliferate, function and survive in hostile tumor settings. Here costimulatory switch proteins (CSP) that turn negative signals into positive signals are at the forefront of development of next generation TCR-T therapies (141, 142). Two CSP are developed in the E2E platform to armor and/or enhance the functional capacities of TCR-T cells to directly attack tumor cells and to reshape the TME. These chimeric proteins are integrated into the same transfer vector, alongside a rTCR that directs the TCR-T cells to their primary cellular targets of importance, which express the relevant pHLA complex specific for the 3S TCR.

### Armoring TCR-T cells and enhancing their functions with a PD1-41BB CSP

One very prominent immunosuppressive pathway in T cells used by tumor cells for counter-attack is mediated through the PD-1/PD-L1 inhibitory axis (143–146). Engagement of PD-1 receptors on T cells by PD-L1 ligands on tumor cells transmits a signal in T cells that inhibits proliferation, cytokine production, and killing. Many cancers upregulate PD-L1, which is even enhanced by IFNγ secretion by T cells upon tumor cell engagement. PD-L1 is deployed by tumor cells to cripple naturally occurring T cell-mediated antitumor responses. Likewise, TCR-T cells are negatively controlled by PD-L1 expression by tumor cells (83, 146).

TCR-T cells also face major challenges to sustain function upon repetitive exposure to tumor cells. Optimal T cell activation is initiated via interactions with professional antigen-presenting cells, such as DC, that deliver signals to TCR and also provide costimulatory signals to amplify T cell activation (147, 148). Without costimulation, T cell proliferation and functional activity does not reach full capacity and is not sustained; rather T cells become exhausted and even driven into apoptosis when they receive TCR signals in the absence of costimulation. A dramatic impingement of TCR-T cell function is observed by repetitive exposure to tumor cells (83, 146). This occurs because the tumor cells express pHLA ligands that signal the TCR but they lack ligands required for T cell costimulation, via the CD28 or CD137 (41BB) pathway (146, 148, 149). As a result, repetitive exposure of TCR-T cells leads to metabolic stress and exhaustion (83, 148, 149). Thereby, tumor cells cause demise of TCR-T cell function through two combined mechanisms – signaling of rTCR by tumor cell-associated pHLA complexes in the absence of costimulation and engagement of the PD-1 inhibitory pathway through expression of PD-L1.

**Figure 14:**
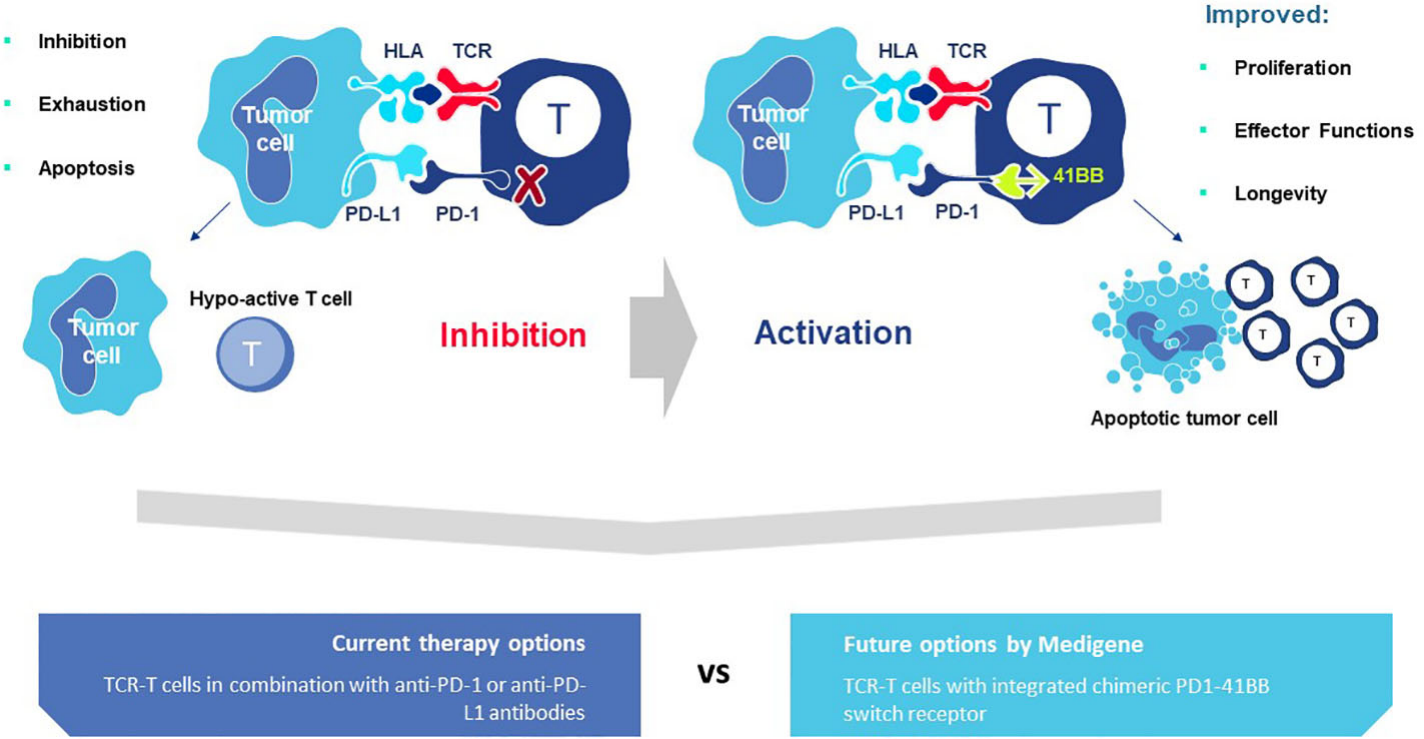
PD1-41BB Switch Receptor converts inhibition to activation of TCR-T cells. TCR-T cells encounter two major impediments in the TME of many solid cancers. Tumor cells can express pHLA ligands that stimulate rTCR on TCR-T cells, but in the absence of co-stimulation through the CD28 or CD137 (41BB) pathways, persistent TCR signaling leads to loss of function, exhaustion and eventually also to cell death by apoptosis. This inhibition is further amplified if the tumor cells express PD-L1 which binds to PD-1 receptors on activated TCR-T cells and induces an inhibitory signaling pathway in T cells that also drives them to become hypoactive. The PD1-41BB Switch Receptor is designed to overcome both of these impediments to TCR-T cell function in the TME. TCR-T cells are engineered to express a rTCR that recognizes a target pHLA ligand on tumor cells and upon binding transmits an activation signal in the TCR-T cells. At the same time, the extracellular domain of the co-expressed PD1-41BB switch receptor interacts with PD-L1 on tumor cells and delivers a second activating signal, instead of an inhibitory signal, to the TCR-T cells through the intracellular 41BB co-stimulatory pathway. When both receptors are engaged by tumor cells, the TCR-T cells display dramatically enhanced proliferation, strong functional activation and prolonged survival upon multiple encounters with tumor cells. In this way, two detrimental attributes of tumor cells are turned from inhibition to activation of TCR-T cells (upper figure). It is currently studied whether combination therapies that combine adoptive cell therapy using TCR-T cells to combat solid cancers can be combined with checkpoint inhibitor antibodies that block the PD-1/PD-L1 pathway, to overcome inhibition and improve TCR-T therapeutic efficacy (lower left). The intrinsic combination of the rTCR with the PD1-41BB switch receptor provides a 2-in-1 combination therapy that changes inhibition into activation and simultaneously overcomes two major impediments to TCR-T cell function, potentially improving therapeutic efficacy (lower right).

The E2E Platform provides a highly innovative technology to overcome TCR-T cell deficiencies caused by these dual mechanisms of tumor suppression. This is based on use of a proprietary *PD1-41BB CSP* that is integrated in recipient T cells that are also engineered to express a 3S TCR (**Figure 14** upper). The natural external PD-1 domain of the PD1-41BB switch receptor binds to PD-L1 on tumor cells, but instead of causing inhibition, it amplifies T cell functions through activation of the 41BB costimulatory pathway in the T cells via the intracellular domain of the CSP. In this way, a strong inhibitory signal is changed to an activating signal in the TCR-T cells. TCR-T cell activities can be further fostered by sequential confrontation with tumor cells that express pHLA complexes binding the rTCR and PD-L1 binding to the CSP (83, 146). This approach provides a very simple mechanism to dramatically change the dynamics of TCR-T cell responses to tumor cells. Other variations of switch proteins are in development for use in TCR-T therapies that utilize different external binding domains or internal signaling pathways to mobilize cytokine production or amplify multiple effector cell activities in TCR-T cells (149–152).

One major advantage of the PD1-41BB CSP is that it ties together two pathways that are well known and well understood for their impact on positive and negative regulation of T cell function. The 41BB internal signaling domain has been used extensively in CAR-T therapies where it has been found to be important in controlling T cell proliferation and persistence (153, 154). 41BB is a well-studied pathway of costimulation that is prominent in CD8 T cells. The blockbuster success of checkpoint inhibitor antibodies interfering in the PD-1/PD-L1 axis is also well known in cancer immunotherapy (155–157). The PD1-41BB CSP profoundly impacts TCR-T cell function, as demonstrated both *in vitro* and *in vivo*, through stronger proliferation, diverse poly-cytokine secretion, improved metabolic function, maintenance of stemness and enhanced killing capacity, which even improves through serial exposure to tumors^13^ (83, 146). It redeploys a major inhibitory mechanism used by tumors to escape immune control and instead causes tumor cells to drive their own destruction. A major advantage is achieved through the co-expression of the rTCR and the PD1-41BB switch receptor in the same TCR-T cells. The integration of these two elements in the same T cells creates an intrinsic 2-in-1 combination therapy with the potential to eliminate the need to combine TCR-T therapy with an extrinsic antibody to block PD1/PD-L1 inhibition (**Figure 14**, lower).

### Enhancing TCR-T function and reshaping the TME with a CD40L-CD28 CSP

The influence of the CD40/CD40L axis extends far beyond its impact on the tumor vasculature and leukocyte trafficking, as it also modulates the activities of many other cellular components in the TME (121, 142). Many tumors express CD40 and engagement with T cells expressing CD40L can trigger tumor cell apoptosis. This can occur through both HLA-restricted and HLA-independent recognition. TAM that express CD40 can switch their activities from immunosuppression to tumor killing after interaction with CD40L^+^ T cells that secrete Th1 cytokines. CD40L^+^ CD4 T cells activate DC through CD40, giving them license to prime CD8 T cells by presentation of pHLA ligands and upregulation of CD80 and CD86 molecules that provide costimulatory signals to T cells expressing CD28 receptors. CD28 activation in T cells leads to strong proliferation and secretion of cytokines, of which IL2 is very prominent. Activated DC produce IL12 that fosters further immune cell recruitment and activation of NK cells that kill tumor cells that lose HLA expression and are no longer recognized by T cells. This multitude of changes dramatically reshapes the TME, enabling multiple cellular players to mediate antitumor immunity.

To capture the diversity of response that can be modulated by the CD40/CD40L axis, the E2E Platform includes a highly innovative CSP that fuses the extracellular CD40L domain with the intracellular signaling domain of CD28 (142). When our proprietary *CD40L-CD28 CSP* is co-expressed with a rTCR specific for a pHLA ligand on tumor cells, four important attributes are displayed by the TCR-T cells: activation of endothelial cells to allow trans-migration of immune cells, activation of TAM, activation of intra-tumoral DC and killing of CD40^+^ tumor cells. All of these enhancing functions can be attributed to the CD40L extracellular domain of the CSP. Furthermore, the TCR-T cells show strong proliferation, secretion of IL2, and activation of DC to secrete IFNα and IL12. These enhancing activities are dependent on signaling through the TCR which enables the CD28 pathway to provide the TCR-T cells with costimulation subsequent to CD40L interaction with CD40^+^ cells. Thus, addition of the CD40L-CD28 CSP to TCR-T cells provides them with multiple new attributes that are projected to improve TCR-T cell trafficking, broaden antitumor responses through DC activation and shape the TME to be more conducive to antitumor immune responses.

The use of chimeric proteins to armor and enhance multiple mechanisms in TCR-T therapy should strongly improve their potential to control and eradicate cancer cells, in analogy to the tumor control seen in animal models using such fusion proteins *in vivo*, providing potentially more efficacious treatments for patients with strongly inhibitory TME (83, 146). These intrinsic combinations simplify clinical development since assessment of safety, tolerability and dosing is made with a single drug product that nevertheless mobilizes multiple modes of action of two-component combination therapies. This can foster faster clinical development of TCR-T therapies to the ultimate benefit of patients, with potentially lower toxicity and lower cost as well.

## Discussion

Nature has invested more than 300 million years in the evolution of T cells as drivers of human immune defense against pathogens and cancer. T cell dominance in cancer defense derives from the capacity of T cells to directly recognize and kill aberrant tumor cells in a highly specific manner while not attacking normal healthy tissues, in most instances. As such, leveraging the extensive benefits of evolution in deriving natural TCR from healthy donors, has significant potential benefits, particularly from a safety perspective. In addition to killing mechanisms, T cells secrete diverse chemokines and cytokines upon antigen-specific stimulation that amplify their activities against tumor cells and enable them to recruit additional players into a complex network of cellular defense. Studies of the TME now reveal important mechanisms of immune amplification led by T cells that recruit innate and other adaptive cells to fight cancer masses comprised of heterogenous populations of cells. At the same time, TME studies identify counter-mechanisms used by tumor cells to create formidable barriers for effective T cell attack. Such counterpoints occur at the level of individual tumor cells and by cancer masses in aggregate, which include other immunosuppressive cell types. Elucidation of mechanisms that govern tumor cell-T-cell interactions in the TME allow new approaches to improve patient antitumor responses to be developed. In particular, implementation of synthetic biology can be used to create better T cells for patients using chimeric proteins to embody TCR-T cells with new capabilities that rely upon modulation of natural pathways of T cells, as exemplified by “evolution by innovation” displayed in the E2E Platform.

TCR-T cells can be modified to express rTCR with higher-affinity for sensitive cancer cell recognition but still stay within the natural bounds of TCR-pHLA interactions through use of non-mutated TCR variable regions. This conservation, in turn, dictates that natural on-off rates of TCR engagement occur with their pHLA ligands, which titrate T cell function and allow T cells to mediate serial killing. Synthetic biology can be applied, however, to overcome limitations in TCR-T functional avidity by improving constant region pairing to increase levels of TCR surface expression which still remain within natural limits set by the available amounts of CD3 (158, 159). Thus evolution by innovation can improve TCR-T functions via their TCR structures but still utilize mechanisms of restraint set by nature, as developed with Precision Pairing. Alternatively, synthetic TCR-T cells can be developed that introduce a new method of T cell control via an inducible iM-TCR that is designed to tame natural activities of T cell responses that can become highly detrimental when T cell driven inflammation cannot be well accommodated for cancer control, if it is not finely tuned. Hereby, TCR-T cell therapies can be tailored to diminish over-shooting responses for treatment of indications, like brain tumors, where natural, but incessant, T cell-driven inflammation could preclude therapeutic options for patients.

Going beyond the rTCR structure itself, synthetic biology can be applied to recipient T cells to improve a panoply of effector functions in TCR-T cells that contribute to optimal antitumor responses. PD-1/PD-L1-mediated inhibition of T cells underpins the use of therapeutic antibodies to block the PD-1/PD-L1 axis in order to reanimate activities of TIL present in the TME. Such antibody therapies are most effective in TME that are rich in T cell infiltrates and display pro-inflammatory characteristics supportive for amplification of antitumor responses. These are described as “hot” tumors, while tumors with a paucity of TIL are classified as “cold” if their TME do not display pro-inflammatory characteristics. There are gradations in between these two extremes (160–163). It is envisioned that adoptive transfer of TCR-T cells can compensate for missing TIL, providing active cytotoxic T cells capable of specific tumor cell killing and also deliver cytokine mediators that alter the TME to become more pro-inflammatory and support further amplification of antitumor responses, as is envisioned with the CD40L-CD28 CSP. However, as with natural TIL, tumors initiate counter-measures against TCR-T cells, with the PD-1/PD-L1 inhibitory axis serving as one prominent mechanism. For this reason, new trials of TCR-T therapy move to incorporate antibodies to block the PD-1/PD-L1 axis in combination treatments (7–9).

TCR-T therapy combined with checkpoint antibodies as two separate therapeutic modalities, will very likely improve treatment success but may also lead to higher levels of toxicity in patients, with particular impact coming from the known toxicities associated with systemic distribution of checkpoint inhibitor antibody, even though TCR-T cell functions may remain localized to the TME through TCR-dictated recognition of tumor cells. Alternative approaches for intrinsic combination therapies are now explored that remain localized to the TME by directly building them into TCR-T cells, as exemplified through switch receptor technologies or membrane-bound cytokines (150, 156, 157). The PD1-41BB CSP technology is developed to locally overcome tumor-mediated inhibition by PD-L1, while simultaneously employing 41BB-induced costimulation to amplify many different functional capacities of TCR-T cells. This represents a dual armoring and enhancement combination. The CD40L-CD28 CSP provides a dual combination of enhancements: one mediates signals to the outside to shape the TME and the other provides signals to the inside to amplify proliferation and function of the TCR-T cells themselves. Once again, evolution by innovation utilizes sophisticated synthetic biology to engineer TCR-T cells to mobilize a panoply of natural T cell effector mechanisms that are used to fight cancer cells directly and to alter the TME through integration of additional players in a fulminant immune response. Both CSP of the E2E Platform use the normal extracellular and transmembrane domains of their counterpoint proteins which dictate natural on-off rates of interaction with their interacting partners and maintain the spatial orientation on the TCR-T cell as it is deployed in nature. The delivery of intracellular activation signals to the T cells mobilizes natural pathways of T cell costimulation that remain under normal control by the adaptor proteins that naturally regulate these pathways (153, 154), since the 41BB and CD28 domains are not engineered in any way to hinder this form of regulation. Thus, these CSP hone closely to nature for regulated external and internal interactions, providing technologies that are likely to be beneficial in TCR-T therapies without unwarranted toxicity. This is indicated by a similar approach applied in a CAR-T therapy employing an alternative switch protein using PD-1 combined with CD28 signaling (164, 165).

**Figure 15:**
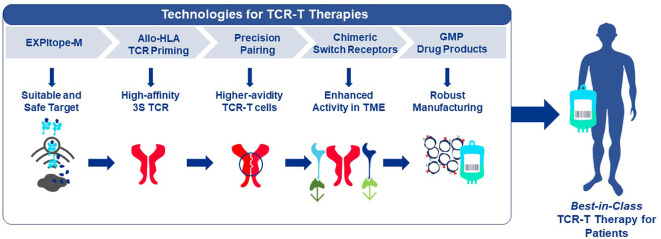
Evolution by Innovation: Connecting the Dots for TCR-T Therapies. The E2E Platform is designed to evolve by innovation through inclusion of new technologies to improve clinical efficacy and safety of TCR or to improve developmental processes across the spectrum of activities needed to develop TCR-T therapies. Multiple, combinable, innovative technologies can be employed in a sequential manner. An example is illustrated showing how five individual technologies across the platform can be combined to provide best-in-class TCR-T therapies for patients in the future.

The different innovative technologies in the E2E Platform described here exemplify our concept of evolution by innovation that is implemented in a stepwise and sequential manner to improve TCR-T therapies. These innovations can be used singly or in combination, as dictated by the state of implementation and clinical need. A future TCR-T therapy encompassing multiple innovations is illustrated in **Figure 15**, as an example of the driving force in the E2E Platform applied across multiple modules to support the goal of developing differentiated, best-in-class TCR-T therapies as new and better treatment options for cancer patients. In its current configuration, the E2E Platform directly addresses the major impediments illustrated in **Figure 1** and uses technological innovation to improve the potential of TCR-T therapies to offer greater clinical benefit to patients.

## Author contributions

The author confirms being the sole contributor of this work and has approved it for publication.

## Footnotes

1
https://medigene.de/wp-content/uploads/20230406-EBMT-P227_MDG.pdf

2
https://medigene.com/fileadmin/download/abstracts/20221108_Medigene_Cell_UK.pdf

3
https://www.adaptimmune.com/investors-and-media/news-center/press-releases/detail/237/.

4
https://www.atcc.org/.

5
https://www.culturecollections.org.uk/collections/ecacc.aspx.

6
https://nanostring.com/products/ncounter-analysis-system/ncounter-pro/.

7
https://medigene.com/fileadmin/download/publications/20201207:MDG_ASH_Poster_Screening-Study.pdf.

8
http://webclu.bio.wzw.tum.de.expitope.

9
https://alithea-bio.com/.

10
https://medigene.com/fileadmin/download/publications/191030_Poster_Neo_SITC_DSo.pdf.

11
https://medigene.com/fileadmin/download/publications/OgonekJustynaPoster189.pdf.

12
https://medigene.com/fileadmin/download/publications/20211027:Medigene_Neoantigen-Summit.pdf.

13
https://medigene.com/wp-content/uploads/20230329-Boston-TCR-Summit_Schendel_Final.pdf.

14
https://medigene.com/fileadmin/download/publications/20211208:ESMO-IO_Medigene-iM-TCR_52P-Andreas-Acs.pdf

15
https://medigene.com/wp-content/uploads/MDG-Corporate-deck-Apr-1-2023-Update-final-1.pdf.

## Glossary

**Table:**

ACT	adoptive cell therapy
AML	acute myeloid leukemia
CAR-T	Chimeric antigen receptor-T
CD	cluster of differentiation 3, 4, 8, 28, 40, 62E (also E-selectin) 80, 86, 137 (also 4-1BB)
CD40L	CD40 ligand
CEA	carcinoembryonic antigen
CGA	cancer-germline antigen
CSP	costimulatory switch protein
CTA	cancer-testis antigen
E2E	end-to-end
DC	dendritic cell
EBV	Epstein-Barr Virus
ER	endoplasmic reticulum
Fas	FS-7 associated surface antigen
FasL	Fas ligand
GvHD	graft-versus-host disease
HLA	human leukocyte antigen
HSCT	hematopoietic stem cell transplantation
HTS	high-throughput screening
ICAM-1	intracellular adhesion molecule-1
IFN-γ	interferon-gamma
IL2	interleukin 2
IL12	interleukin 12
ivtRNA	*in vitro* transcribed ribonucleic acid
MAGE	melanoma-associated antigen
mHA-1	minor histocompatibility antigen 1
MHC	major histocompatibility complex
MM	multiple myeloma
MDS	myelodysplastic syndrome
MS	mass spectrometry
MDSC	mesenchymal-derived suppressor cell
NGS	next generation sequencing
NK	natural killer
NY-ESO-1	New York esophageal-1
PD-1	programmed cell death protein 1
PD-L1	programmed cell death-ligand 1
pHLA	peptide-human leukocyte antigen
rTCR	recombinant T cell receptor
TAA	tumor-associated antigen
TAM	tumor-associated macrophage
TAN	tumor-associated neutrophil
TCE	T cell engager
TSA	tumor-specific antigen
TAP	transporter associated with antigen processing
TCR	T cell receptor
Th1	T helper 1
Treg	T regulatory cell
TIL	tumor-infiltrating lymphocytes
TME	tumor microenvironment
TNF-α	tumor necrosis factor alpha
VCAM-1	vascular cell adhesion molecule-1
WT1	Wilms’ tumor 1
